# Analysis of genomic distributions of SARS-CoV-2 reveals a dominant strain type with strong allelic associations

**DOI:** 10.1073/pnas.2007840117

**Published:** 2020-11-12

**Authors:** Hsin-Chou Yang, Chun-houh Chen, Jen-Hung Wang, Hsiao-Chi Liao, Chih-Ting Yang, Chia-Wei Chen, Yin-Chun Lin, Chiun-How Kao, Mei-Yeh Jade Lu, James C. Liao

**Affiliations:** ^a^Institute of Statistical Science, Academia Sinica, Taipei 11529, Taiwan;; ^b^Department of Statistics, Tamkang University, New Taipei City 251301, Taiwan;; ^c^Biodiversity Research Center, Academia Sinica, Taipei 11529, Taiwan;; ^d^Institute of Biological Chemistry, Academia Sinica, Taipei 11529, Taiwan

**Keywords:** COVID-19, mutation, single nucleotide variation, allelic association, sequencing

## Abstract

Severe acute respiratory syndrome coronavirus 2 (SARS-CoV-2), the causal agent of COVID 19, continues to evolve since its first emergence in December 2019. Using the complete sequences of 1,932 SARS-CoV-2 genomes, various clustering analyses consistently identified six types of the strains. Independent of the dendrogram construction, 13 signature variations in the form of single nucleotide variations (SNVs) in protein coding regions and one SNV in the 5′ untranslated region (UTR) were identified and provided a direct interpretation for the six types (types I to VI). The six types of the strains and their underlying signature SNVs were validated in two subsequent analyses of 6,228 and 38,248 SARS-CoV-2 genomes which became available later. To date, type VI, characterized by the four signature SNVs C241T (5′UTR), C3037T (nsp3 F924F), C14408T (nsp12 P4715L), and A23403G (Spike D614G), with strong allelic associations, has become the dominant type. Since C241T is in the 5′ UTR with uncertain significance and the characteristics can be captured by the other three strongly associated SNVs, we focus on the other three. The increasing frequency of the type VI haplotype 3037T-14408T-23403G in the majority of the submitted samples in various countries suggests a possible fitness gain conferred by the type VI signature SNVs. The fact that strains missing one or two of these signature SNVs fail to persist implies possible interactions among these SNVs. Later SNVs such as G28881A, G28882A, and G28883C have emerged with strong allelic associations, forming new subtypes. This study suggests that SNVs may become an important consideration in SARS-CoV-2 classification and surveillance.

## Significance

In this study, we discovered that the genome of SARS-CoV-2 to date can be classified in six major types characterized by 14 signature single nucleotide variations (SNVs). In particular, type VI, that was first reported in China and spread to different countries, has become the major type (more than 95% among data collected after mid-May 2020). The signature SNVs for this strain type, C241T (5′UTR), C3037T (nsp3 F924F), C14408T (nsp12 P4715L), and A23403G (S protein D614G), exhibit high pairwise allelic associations, and the haplotype 241T-3037T-14408T-23403G has the highest frequency. Understanding nucleotide variations in the SARS-CoV-2 genome will provide useful insight for the developmental history of the pandemic, and even the disease management, if the biological significance is understood.

Severe acute respiratory syndrome coronavirus 2 (SARS-CoV-2) virus has caused the most significant pandemic (COVID-19) in recent history. Within 7 mo of its emergence in December 2019, the virus has spread to more than 210 countries, and has caused about 500,000 deaths and 10 million reported cases as of June 30, 2020. This virus is a positive-strand RNA virus with a genomic length of about 30,000 (29,903 nucleotides in the reference genome). Mutations are arising constantly; some of them have diluted away, and others have persisted. Some single-nucleotide variations (SNVs) resulting from mutations may increase the fitness of the virus to the environment, while elevating the efficiency of transmission and altering the clinical outcomes. Excluding the 5′ leader and 3′ terminal sequences, the genome contains 11 coding regions for encoding spike glycoprotein (S), envelop protein (E), transmembrane glycoprotein (M), nucleocapsid protein (N), and several open reading frames (ORFs) (ORF1ab, ORF3a, ORF6, ORF7a, ORF7b, ORF8, and ORF10) with various lengths and biological implications. ORF1ab consists of ORF1a partitioned into nonstructural proteins genes nsp1 to nsp11 and ORF1b partitioned into nsp12 to nsp16. ORF1a overlaps with ORF1b in nsp11 and nsp12, the latter of which encodes RNA-dependent RNA polymerase (RdRp), which is the target of Ramdesivir, a potential antiviral drug to COVID-19.

In this study, we first analyzed the genomic sequence data of 1,932 SARS-CoV-2 strains from Global Initiative on Sharing Avian Influenza Data (GISAID), National Center for Biotechnology Information (NCBI) GenBank, and China National Center for Bioinformation (CNCB) (download date: March 31, 2020). We performed both statistical clustering and phylogenetic tree analyses based on the full genomes to discover strain types and identified signature SNVs in each type independent of the dendrogram construction. Subsequently, we analyzed 6,228 SARS-CoV-2 genomes (download date: April 19, 2020) and 38,248 SARS-CoV-2 genomes (download date: June 8, 2020) to validate the strain types and signature SNVs defined by the initial analysis (*n* = 1,932). Using the 6,228 genomes, we characterized the genomic, geographic, and temporal patterns; inferred allelic association; and constructed the emergence history of the key signature SNVs.

## Results

### Tree Dendrogram Construction and Signature SNVs Identification.

To determine the genomic variation features, we initially analyzed the whole-genome sequence data of 1,932 SARS-CoV-2 strains (available as of March 31, 2020) using four phylogenetic dendrograms, including unweighted pair group method with arithmetic mean (UPGMA) (1), neighbor joining (NJ) (2), maximum likelihood (ML) (3), and maximum parsimony (MP) (4), and one hierarchical clustering tree (5) (*SI Appendix*, Fig. S1). The UPGMA and NJ dendrograms were built using the Kimura 2-parameter (6) distance matrix. The ML and MP dendrograms are non−distance-based phylogenetic trees. The nonphylogenetic complete-linkage (5) hierarchical clustering tree was built using a simple matching similarity matrix computed from 2,139 variation sites by comparing with the strain Wuhan-Hu-1 isolated in China (7) as the reference genome.

The five dendrograms define similar types of viral strains (*SI Appendix*, Fig. S1). We first defined six viral types (types I to VI) based on the UPGMA dendrogram with the help of variation structure in the sorted variation matrix map for the 1,932 viral strains (*SI Appendix*, Fig. S2). The typing was further refined based on the branching patterns of the MP dendrogram (*SI Appendix*, Fig. S3).

**Fig. 1.**
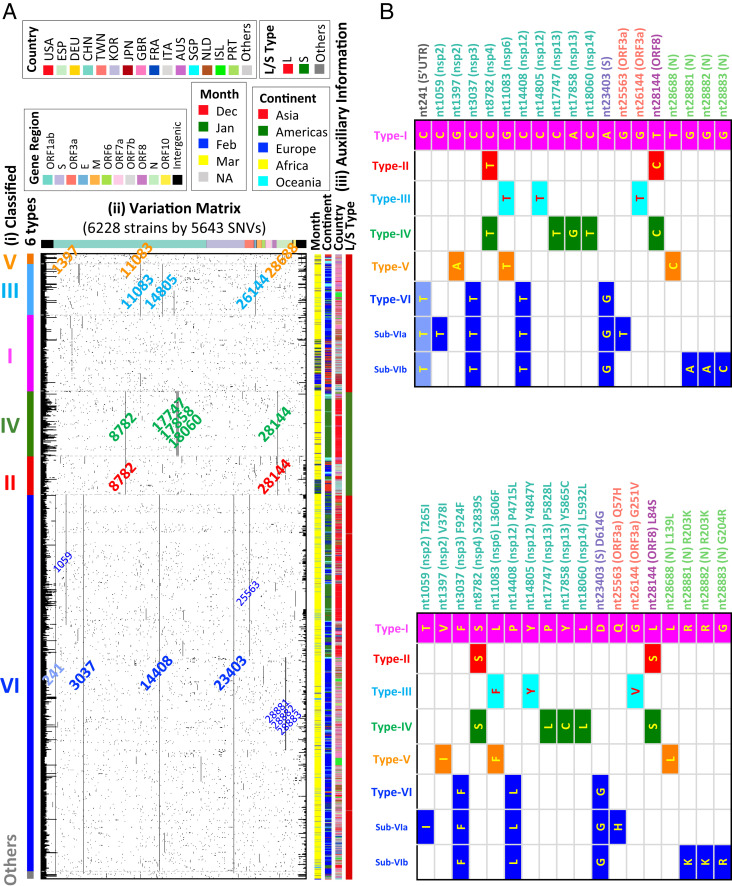
Variation matrix map and viral strain type. (*A*) Classified six types with variation matrix map for 6,228 validated SARS-CoV-2 viral strains. (*i*) Color bar for classified six types for 6,228 validated strains. (*ii*) Variation matrix map for 6,228 strains with 5,643 variation sites. Strains are sorted by classified strain types in the order of V−III−I−IV−II−VI−Others from the MP dendrogram for 1,932 strains. Nucleotides are listed by relative positions in the genome, with color bands indicating their corresponding genome regions on the top. All 5,643 nucleotides have at least one variation among 6,228 strains. Signature variations for each type (and subtypes for type VI) are labeled with corresponding type colors. (*iii*) Auxiliary information for each virus strain on month of data collection, continent, country, two strain types (L, S) defined by Tang et al. (9). (*B*) Annotation of the signature and subtype SNVs. (*Top*) The signature matrix in the level of nucleic acid. (*Bottom*) The signature matrix in the level of amino acid. USA, United States of America; ESP, Spain; DEU, Germany; CHN, China; TWN, Taiwan; KOR, Korea; JPN, Japan; GBR, Great British; FRA, France; ITA, Italy; AUS, Australia; SGP, Singapore; NLD, The Netherlands; ISL, Iceland; PRT, Portugal.

Interestingly, we found 14 signature SNVs in the six viral types, which were independent of the dendrogram construction (Fig. 1 and *SI Appendix*, Table S1) and the reference strain chosen. Since the SNV C241T in the 5′ untranslated region (UTR) has uncertain significance, and the association properties can be represented by the other three strongly associated signature SNVs (Fig. 1*A*), we focus on the other three. Each type was defined by at least two of the remaining 13 signature SNVs, except type I, which carries zero or one of the 13 signature SNVs. The results of the six types of the strains and their underlying signature SNVs were validated in the subsequent analysis of 6,228 genomes downloaded on April 19, 2020 (Fig. 1*A*) and 38,248 strains downloaded on June 8, 2020. More than 98.65% (= 6,144/6,228) and 98.58% (= 37,704/38,248) of strains can be classified into the six types (*SI Appendix*, Table S2) in each of these larger datasets, respectively. We therefore propose to classify the viral strains based on signature SNVs without referring to the phylogenetic or clustering trees.

On the basis of the signature SNVs identified, we developed two efficient algorithms for the strain typing without the need for time-consuming multiple sequencing alignment. One applies a pairwise sequence alignment (8) and another uses a text mining approach. Using the dataset of *n* = 6,228, we found that the pairwise sequence alignment approach exhibited a 60-fold increase in computational efficiency compared to the strain typing relying on multiple sequencing alignment, and the text mining approach showed a 150,000-fold improvement (*SI Appendix*, Table S2). In terms of accuracy, compared to the result based on multiple sequencing alignment, the pairwise sequence alignment approach has an accuracy of 100%, and the text mining approach has an accuracy of >99.6%. The methods were then applied to an even larger genomic dataset of 38,248 strains downloaded on June 8, 2020. More than 98.58% (= 37,704/38,248) of strains can be classified into the six types (*SI Appendix*, Table S2). These results suggest that the proposed six types and 14 signature SNVs are robust.

### Genomic Constitution, Geographic Distribution, and Temporal Progression of Six Strain Types.

**Fig. 2.**
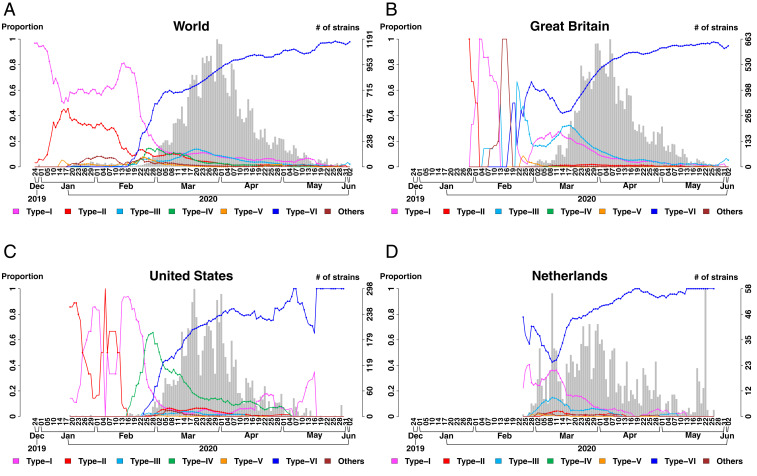
Temporal distributions of the six types. (*A*) The globe; (*B*) Great Britain; (*C*) United States; (*D*) Netherlands. In each plot, the proportions of type I through type VI are displayed using six curves with different colors. The left-hand-side vertical axis indicates the moving-window proportion calculated by dividing the number of the strains belonging to a specific type by the total number of the strains for the samples within four dates of a specific date in each side. The right-hand-side vertical axis indicates the number of strains (i.e., sample size). Sample size is displayed with a histogram in the background.

**Fig. 3.**
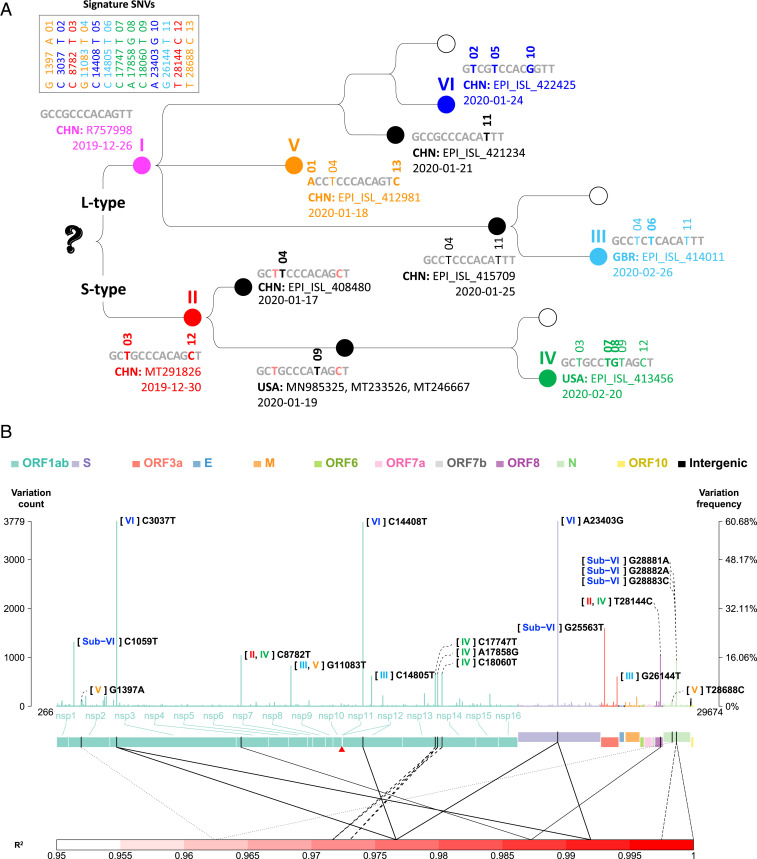
Signature SNVs. (*A*) Emergence history of the 13 signature SNVs in protein coding regions and six types. For each strain type, the signature SNVs, first observation time, country, and strain name are shown. (*B*) Genomic profile of the average variation counts per sample across the viral genome. In each site, the left-hand-side vertical axis indicates the total counts of variations at a site. The right-hand-side vertical axis indicates variation frequency, that is, the average variation counts per sample (i.e., the number of variations that occurred at a site in all viral strains divided by the number of strains). Variations in different gene regions are displayed in different color. A red triangle indicates the starting site of −1 ribosomal frameshift signal in ORF1ab. Two ends (5′ leader and 3′ terminal sequences) are not shown. Pairwise allelic association (R^2^) is shown only for the pairs of the signature and subtype SNVs with an R^2^ value of >0.95, and the same line type is used to indicate a pair of SNVs with strong allelic association.

On the basis of the analysis of 38,248 strains, the proportion of the SARS-CoV-2 strains in the six types is dynamic and changes with time and geographic regions (Fig. 2). The first reported dates and countries of the six types are shown in Fig. 3*A*. Note that the real occurrence date of variations could be earlier because of left censoring of new SNV events. The dates of sample collection of type I and type II were closest to the first emergence of COVID-19 in December 2019. Types I and II were first observed in China on December 26 and December 30, 2019, respectively. These two types were the dominant groups before mid-February 2020 but became the minority groups after March 2020, when type VI took over (Fig. 2*A*). The first two strains which were observed outside of China (in Australia on January 3, 2020 and Thailand on January 5, 2020) belong to type I, illustrating that the international transmission of COVID-19 can be traced back to as early as January 3, 2020. Types III and IV were the only two types for which their first observations were outside of China. Type III was first observed in Great Britain, on February 26, 2020, and type IV was first observed in the United States on February 20, 2020. Type V was first observed in China on January 18, 2020 and represents as a minor population. Type VI was first observed in China on January 24, 2020 and transmitted to other continents and increased its frequency after February 20, 2020 (Fig. 2*A*). Subsequently, type VI was observed in Europe (Italy) on February 20, in Oceania (Australia) on February 22, in Americas (Brazil) on February 25, and in Africa (Nigeria) on February 27. Type VI has become the dominant group in the world since March 2020 (Fig. 2*A*). We also analyzed the distributions of the strain types in the top three nations contributing the most samples: Great Britain (*n* = 18,024) (Fig. 2*B*), United States (*n* = 6,974) (Fig. 2*C*), and Netherlands (*n* = 1,436) (Fig. 2*D*). The results of the other nations that contributed significant sample sizes are also provided (*SI Appendix*, Fig. S4). The increasing trend of type VI was observed in most countries. A few exceptions, such as Iceland and Austria, have a limited sample size after March 2020.

Compared with the reference genome, Wuhan-Hu-1 (7), the average variation counts per sample in types I to VI were 3.99, 6.95, 7.02, 7.12, 7.17, and 7.04, respectively, among 6,228 SARS-CoV-2 strains studied. Among the nations having at least 30 cases reported in this dataset, the top three nations with the highest average variation counts per sample were Spain (7.60), Australia (7.55), and Great Britain (7.43). The three nations with the lowest average variation counts were located in Asia: Japan (3.27), China (3.58), and Singapore (3.90). This phenomenon partially reflected the early occurrence of COVID-19 in Asia.

### Signature SNVs.

The six major types of SARS-CoV-2 strains identified in the clustering dendrogram can be characterized by the 13 signature SNVs in protein coding regions and one SNV in the 5′ UTR (Fig. 1). Their genomic locations in protein coding regions and variation frequencies are displayed in Fig. 3*B*. The signature SNVs for a specific strain type first coobserved in the same strain exhibited strong allelic association (Fig. 3*B*). For example, in type II signature SNVs C8782T and T28144C that were also used to define the S and L type of SARS-CoV-2 (9), these two SNVs (i.e., S type) first coappeared in strain MT291826 on December 30, 2019, and the coefficient of allelic association is R^2^ = 0.987. The reference strain [Wuhan-Hu-1 (7)] used in this study is the L type, while the S type exhibits both new variations. The high allelic association implies that the new variations on the same haplotype were cotransmitted during infection. Other cases first coobserved at the same date included types IV, V, and VI. Two type IV signature SNVs, C17747T and A17858G, first coappeared in strain EPI_ISL_413456 in the United States on February 20, 2020, and the coefficient of allelic association is R^2^(C17747T, A17858G) = 0.973. Two type V signature SNVs, G1397A and T28688C, first coappeared in strain EPI_ISL_412981 from China on January 18, 2020, and the coefficient of allelic association is R^2^ = 0.962. Type VI signature SNVs C3037T, C14408T, and A23403G (abbreviated as CCA for the reference genome and TTG for type VI) first coappeared in strain EPI_ISL_422425 from China on January 24, 2020, and the pairwise coefficient of allelic association is R^2^(C3037T, C14408T) = 0.977, R^2^(C3037T, A23403G) = 0.992, and R^2^(C14408T, A23403G) = 0.977. There is another SNV in type VI (C241T in the 5′ UTR) that is in strong allelic association with the three signature SNVs (10). Since this SNV is in the UTR, of which the role is still unclear, we focus our attention on the other three SNVs. The temporal profiles of allelic association R^2^ showed that allelic association among type VI signature SNVs attenuated and then recovered to strong allelic association (*SI Appendix*, Fig. S5; the R^2^ values based on the daily data [red dotted curve] and the cumulative data [black dotted curve]). This illustrated that, in addition to the reference haplotype CCA and the current dominant haplotype TTG, a few additional haplotypes had occurred in the developmental history of the SARS-CoV-2 strains but did not persist.

Note that the signature SNVs of types II, V, and VI were first coobserved in the same strain on the same lineage on December 30, 2019, January 18, 2020, and January 24, 2020, respectively, and they exhibited strong allelic associations within each type. In contrast, type III signature SNVs G11083T, C14805T, and G26144T first occurred sequentially in different strains on different dates (Fig. 3*A*), and they exhibited relatively low pairwise allelic associations—R^2^(G11083T, C14805T) = 0.469, R^2^(G11083T, G26144T) = 0.553, and R^2^(C14805T, G26144T) = 0.723. These examples illustrate that allelic associations may reflect the concurrent or sequential occurrence of signature variations and improve the understanding of the emergence of the strain types.

In addition, the signature SNVs with high allelic association are typically located in distant regions, except for type IV signature SNVs C17747T and A17858G and the sub-VI SNVs (refer to the next section and Fig. 3*B*) G28881A, G28882A, and G28883C. This phenomenon is different from the species with historical recombination, such as *Homo sapiens*. In *Homo sapiens* populations, intermarker allelic association (also called “linkage disequilibrium”) is typically observed in neighboring regions and reflects local proximity and low recombination fraction among neighboring markers in a genomic region. We recognize that GISAID has also identified a set of signature SNVs, which overlap with those we identified previously (10) and reported here.

### The Dominance and Persistence of Type VI Signature SNVs.

**Fig. 4.**
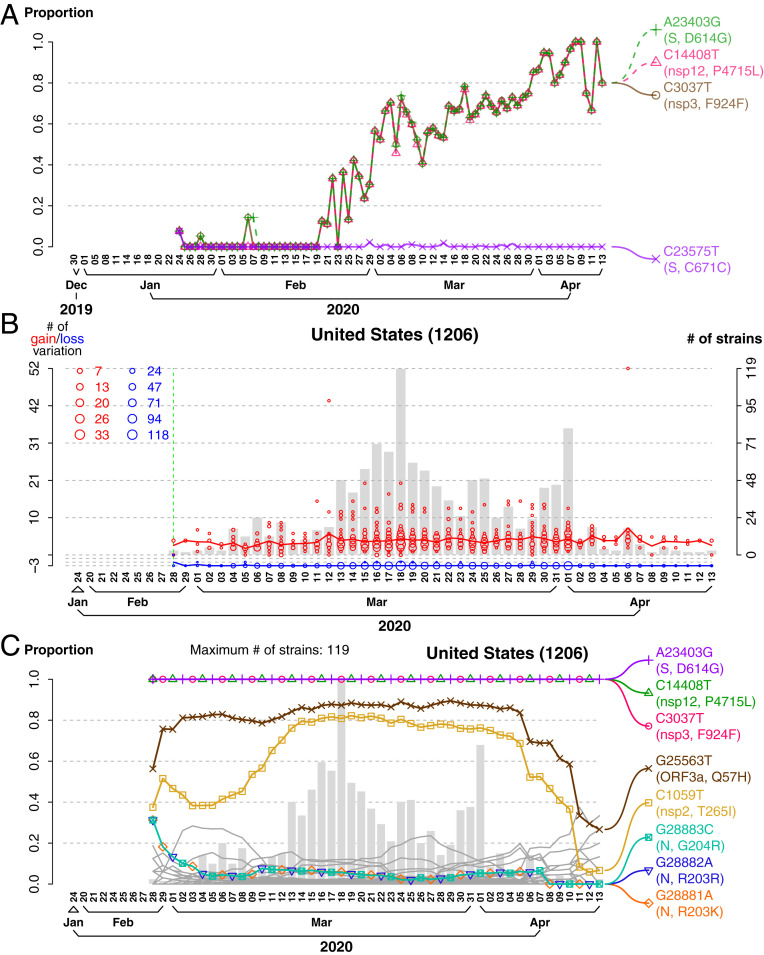
Dominance and persistence of type VI signature SNVs. (*A*) Temporal proportion of the new variations first coobserved in the strain EPI_ISL_422425 from China on January 24, 2020. Average variation counts per sample for C3037T (F924F), C14408T (P4715L), A23403G (D614G), and C23575T (C671C) first coobserved in EPI_ISL_422425 in China are displayed since January 24, 2020. The TTG signature SNVs persisted, but C23575T was lost immediately in the samples. (*B*) Number of variation gain and loss in the type VI strains in the United States. The left-hand-side vertical axis indicates the numbers of variation gain and loss. We picked one at random from the strains in the first date of sample collection in the United States as a reference strain. Compared with the first strain in the United States, in addition to the TTG signature SNVs, the type VI strains in the United States had, at most, three additional variations. Blue circles indicate the number of strains that lost the additional variations. Red circles indicate the number of strains that gained variations not in the reference strain. The larger circle represents the larger number of strains that lost/gained the additional variations. Sample size is displayed with a histogram in the background. (*C*) Temporal frequency of the variations in the type VI strains in the United States. The moving-window proportion on a sample collection date was calculated by dividing the number of variations (e.g., allele G for A23403G) by the number of strains within four closest sample collection dates on each side. Sample size is displayed with a histogram in the background. Note that the variations in this figure were those newly added variations compared to the reference strain from the United States (red circles in *B*).

As of June 2, 2020, type VI has become the dominant type in most countries (Fig. 2 and *SI Appendix*, Fig. S4). We therefore focused on the emergence and the progression of this type and found the following features. First, the larger dataset of 6,228 strains showed that the three signature SNVs of type VI occurred simultaneously, on January 24, 2020 (*SI Appendix*, Fig. S6), with another SNV C23575T (S protein, C671C) in the same strain EPI_ISL_422425 in China (Fig. 4*A*). However, the TTG signature SNVs persisted, but C23575T was lost immediately in the samples. This additional C23575T SNV reappeared in different strains occasionally in later samples (Fig. 4*A*), suggesting against the possibility of sequencing error. Remarkably, strains missing one or two type VI signature SNVs were also observed in cluster infection [e.g., in Germany (11)], identified by various sequencing technologies including Illumina, Oxford Nanopore, Ion Torrent, Sanger, and PacBio, and had no significant bias among sequencing technologies. These lines of evidence further support that the finding was not a sequencing artifact. After the emergence of the TTG signature SNVs, there were some cases where one or two SNVs among the TTG signature were lost (*SI Appendix*, Fig. S6). However, these cases did not persist either (*SI Appendix*, Fig. S7). In the dataset of 6,228 strains, 3,745 type VI strains carry the TTG signature SNVs; only 2, 25, and 3 strains carry two variations (3037T, 14408T), (3037T, 23403G), and (14408T, 23403G), respectively; only 4, 5, and 5 strains carry one of the three signature SNVs. These results suggest a much stronger fitness gain of the strains that simultaneously carry the three TTG signature SNVs. Furthermore, to test the statistical significance of the growing popularity of the TTG signature SNVs in time, sample collection dates were randomly permuted 10,000 times. Results showed that the growing haplotype frequency of the TTG signature SNVs was significant for the global data (*P* < 1.00 × 10^−4^) and data in many countries such as the United States (*P* < 1.00 × 10^−4^), Great Britain (*P* < 1.00 × 10^−4^), Australia (*P* = 3.00 × 10^−4^), and France (*P* < 1.00 × 10^−4^).

Second, we used the initial type VI strain in each country as the reference, which typically has more SNVs than the TTG signature SNVs, and examined the loss and gain of additional SNVs in the type VI strains in different countries. Interestingly, the nonsignature SNVs in the initial type VI strain in each country were lost rapidly. For example, in the United States, the initial type VI strains had three SNVs in addition to the signature SNVs, but these additional SNVs were quickly lost across the sample collection dates (blue circles in Fig. 4*B*). Furthermore, up to 52 additional SNVs occurred in type VI strains in the United States (red circles in Fig. 4*B*), but most of them disappeared, except for the five subtype SNVs (Fig. 4*C*). Similar results were also found in many other countries (*SI Appendix*, Fig. S8). The results illustrate the persistence of the three type VI signature SNVs TTG.

Third, the subtypes in the dominant strain (type VI) were seen in the clustering dendrogram and signature SNVs (Fig. 1). In addition to the three type VI signature SNVs, five additional subtype SNVs with variation frequencies of >0.1 included C1059T (nsp2, T265I), G25563T (ORF3a, Q57H), G28881A (N, R203K), G28882A (N, R203K), and G28883C (N, G204R) (Fig. 1 and *SI Appendix*, Table S1). We observed the time trajectory of average variation count for these sub-VI SNVs (*SI Appendix*, Fig. S9). There are two sub-VI groups: VIa and VIb. In addition to the three type VI signature SNVs, VIa carries signature SNVs C1059T and G25563T and was first coobserved in France (EPI_ISL_418218) on February 21, 2020, and the coefficient of allelic association R^2^ = 0.735. VIb carries signature SNVs G28881A, G28882A, and G28883C in strong allelic association and was first coobserved in Germany (EPI_ISL_412912) on the same date, February 25, 2020, and the coefficient of pairwise allelic association is R^2^(G28881A, G28882A) = 0.997, R^2^(G28881A, G28883C) = 0.997, and R^2^(G28882A, G28883C) = 1. The proportion of VIb signature SNVs G28881A, G28882A, and G28883C in type VI (38.56%) has exceeded the proportion of VIa signature SNVs C1059T and G25563T (34.20%). The strains carrying sub-VI SNVs may evolve to new major types in the future.

Globally, type VI is by far the most frequent strain among genomes reported daily since the beginning of March 2020 (Fig. 2*A*). Cumulatively, TTG signature SNVs for type VI had a haplotype frequency of 59.97% among all of the reported genomes in the dataset of *n* = 6,228. The frequency has greatly exceeded the frequency in strains without any of the 13 signature SNVs (9.23%) in protein coding regions. Importantly, the frequencies of haplotype TTG for type VI has increased and become the highest in most countries reporting genome sequences (*SI Appendix*, Fig. S10), suggesting a strong fitness gain in the local environment. This phenomenon is difficult to explain simply by a random drift or the founder effect due to international travel followed by lockdown.

Among the TTG signature SNVs, C14408T (nsp12, P4715L) is located in the RdRp gene that plays a role in replication, and A23403G (S protein, D614G) impacts the S protein that plays a role in receptor binding, membrane fusion, and virus entry. Interestingly, C3730T (nsp3, F924F) is a synonymous mutation in nsp3. If C3730T contributes to the fitness gain, it must be at the RNA level. The SNV (C241T) in the 5′ UTR in type VI is also in strong allelic association with the three signature SNVs (10). Since the 5′ leader sequence region accommodates many variations, we excluded this and the 3′ end in classification. Nevertheless, the significance of C241T SNV deserves further investigation.

We examined the selective pressure in RdRp (from nucleotide site 13,468 to 16,236) and S (from site 21,563 to 25,384). For RdRp, among the six types, only type VI showed a positive value of nonsynonymous substitutions per nonsynonymous site minus synonymous substitutions per synonymous site (dN – dS) (dN – dS = 0.000308, dN/dS = 2.798, *P* = 3.38 × 10^−225^). For S, among the six types, only type VI showed a positive value of dN – dS (dN – dS = 0.000261, dN/dS = 3.375, *P* = 1.68 × 10^−303^). The strong significance was also validated by permutation tests with 100,000 permutations (*P* < 0.00001). Among the six types, only type VI can be claimed as a strain type with significant positive selection in the RdRp and S protein regions after adjusting for multiple testing. With the above results, a pure founder effect due to international travel and lockdown of countries cannot easily explain the dominance and persistence of type VI and the rising haplotype frequency of TTG.

## Discussion

In this study, we first analyzed 1,932 reported genomes of SARS-CoV-2 using four phylogenetic dendrograms and one hierarchical clustering tree. These analyses revealed six major types of SARS-CoV-2 strains, which can be characterized by 14 signature SNVs. This classification was subsequently validated using larger datasets of 6,228 (download date: April 19, 2020) and 38,248 (download date: June 8, 2020). The signature SNVs can classify more than 98% of the strains with complete genomes in the dataset of 38,248. The 14 signature SNVs mostly occurred in ORF1ab, with notable exceptions in S, N, ORF3a, and ORF8. This result suggests the importance of these SNVs in viral fitness and perhaps clinical relevance. In particular, type VI characterized by a triplet (TTG) of signature SNVs has been the dominant strain type (43.7% in the dataset of *n* = 1,932, 60.1% in *n* = 6,228, and 72.5% in *n* = 38,248), and continues to rise in frequency.

Recent studies suggested that D614G in the S protein, caused by one of the signature SNVs (A23403G) in type VI, may increase infectivity of SARS-CoV-2 (12, 13). However, in this study, we found that strains carrying the single SNV (A23403G) but not the other two signature SNVs (C3037T and C14408T) did not persist. This illustrates that the study focusing on D614G alone may be insufficient and must jointly consider other signature SNVs of type VI in order to reach a better understanding regarding the prevalence of COVID-19. This also explains that the strong fitness of the type VI signature SNVs is hard to explain by a genetic hitchhiking with A23403G. In addition, the strong allelic association among the long-distant variations C3037T (nsp3, F924F), C14408T (RdRp, P4715L), A23403G (S protein, D614G), and C241T (5′ UTR) (10) suggest a possible beneficial interaction either among the proteins or RNA regions/species.

Compared to the L and S types originally reported (9), and the A, B, C types reported subsequently (14), our classification of six types provides a variation-based taxonomy of viral strains and explains the heterogeneity of strains within each of L and S types and A, B, C types. Interestingly, five out of six types are characterized by a few signature SNVs in each type. This concept and method for classification and characterization of viral strains can be applied to other viruses of public health concern. As more whole-genome sequencing data of SARS-CoV-2 become available online, we will be better posed to decipher and understand genomic, geographic, and temporal distributions of viral variations. However, representativeness and small sample size in some time periods of the submitted genomic data should be considered carefully when explaining the results.

Interestingly, many signature SNVs first occurred simultaneously, and persist with the coefficient of allelic association close to 1. The simultaneous occurrence may arise because of strong positive interactions at the protein or RNA level, or the SNVs might occur sequentially but appeared simultaneously due to insufficient sampling. The persistence of the signature SNVs may imply a fitness gain or simply a founder effect. However, the multiple lines of evidence presented here favor a positive selection. Nevertheless, the biological implication of each variation and their interactions remain an interesting topic to be explored.

The potential second wave of COVID-19 has been under development in China and other countries since June 11, 2020. It has caused significant attention regarding the strain type and viral source. We downloaded the whole-genome sequence data of the two SARS-CoV-2 strains (EPI_ISL_469255 and EPI_ISL_469254) collected in Beijing on June 11, 2020. Our efficient strain typing algorithm quickly identified that the two strains are indeed type VI, which is the dominant strain that future treatment and vaccine development should consider. Furthermore, the subtype VIb signature SNVs G28881A, G28882A, and G28883C exhibited strong pairwise allelic association of >0.997 and uprising trend in proportion in the type VI strains (more than 38.56%) in the dataset of *n* = 38,248. The significance of these findings remains to be seen.

## Materials and Methods

We downloaded the whole-genome sequence data from the GISAID database (https://www.gisaid.org/), NCBI GenBank (https://www.ncbi.nlm.nih.gov/genbank/), and CNCB (https://bigd.big.ac.cn/ncov/release_genome) on March 31 2020. After discarding the replicated sequences in the three databases and the sequences with a low quality indicator or no quality information, there remained the complete sequences of 1,938 SARS-CoV-2 genomes, including the Wuhan-Hu-1 reference genome with 29,903 nucleotides. Multiple sequence alignment was performed by using multiple sequence comparison by log-expectation (MUSCLE) (15). We used the strain Wuhan-Hu-1 originally isolated in China (7) as the reference genome and defined nucleotides different from the reference as a variation. The variation matrix consisting of the reference (coded as 0) and variation (coded as 1) at each of ∼30,000 loci for each of 1,938 viral strains was constructed. Generalized association plot (GAP) (16) was used to visualize the variation patterns and identify the outliers in variations and samples. Our signature SNV analysis focused on protein coding regions and excluded the 5′ cap and 3′ noncoding region because of a significant number of gaps. That is, the signature SNV analysis focused on nucleotides from positions 266 to 29,674. It is noteworthy that we observed two nucleotides with a nonnegligible variation frequency relative to their neighboring regions: 1) C241T (5′ UTR) with a variation frequency of 45.5% (and this nucleotide was in high allelic association with C3037T [nsp3, F924F], C14408T [nsp12, P4715L], and A23403G [S protein, D614G]) and 2) G29742T (3′ UTR) or G29742A (3′ UTR) with a variation frequency of 5.3%. We removed four samples with a large deletion in ORF8 (sample EPI_ISL_417518 from Taiwan with EPI_ISL_414378, EPI_ISL_414379, and EPI_ISL_414380 from Singapore), sample EPI_ISL_415435 from Great Britain with a large deletion in ORF1ab, and sample EPI_ISL_413752 from China with a large number of deletions (>300 nucleotides). On April 19, 2020, we downloaded the whole-genome sequence data again. We followed the same procedure of quality control to clean the data and obtained 6,228 SARS-CoV-2 genomes for a validation analysis and the main analysis. On June 8, 2020, we downloaded the whole-genome sequence data again. The purpose was to perform a rapid strain typing without a time-consuming whole-genome multiple sequencing alignment and examine the proportion of the defined strain types in globe and nations. We obtained 38,248 SARS-CoV-2 genomes after removing the duplicate samples shown in CNCB. In the analyses that required only the information of strain types and signature SNVs, the dataset of *n* = 38,248 without a time-consuming multiple sequence alignment was used; otherwise, the genomic data of *n* = 6,228 with a multiple sequence alignment were used.

Average variation counts per sample and/or per locus were calculated for nations and for the data collection time points to study geographic and temporal distributions of variations. Variation frequencies in gene regions were illustrated, and coefficient of allelic association (R^2^) between pairs of nucleotides was calculated by using PLINK (17). Annotation of the signature SNVs and subtype SNVs was collected from CNCB. Variation frequency and haplotype frequency were calculated by using a direct counting method. Phylogenetic tree analysis including UPGMA (1), NJ (2), ML (3), and MP (4) were performed by using molecular evolutionary genetics analysis across computing platforms (MEGA X) (18). For UPGMA and NJ, the Kimura two-parameter model was applied to calculate genetic distance. For MP, Subtree-Pruning-Regrafting algorithm (19) was applied for a tree topology search heuristic. GAP (16) was applied to present the relationship between clustering dendrogram and variations. The dN and dS in a gene region were estimated based on the Li−Wu−Luo model (20) by using MEGA X (18). A *t* test was applied to examine whether the mean of statistic dN – dS over the nucleotides in the gene region of interest was statistically significant positive or negative from zero. A permutation test that randomly flipped the pair of dN and dS in every strain and recalculated the mean of statistic dN – dS and *t* test statistic based on the permuted data was performed for 100,000 replications to calculate an empirical *P* value. Moreover, Bonferroni correction was performed to adjust for multiple testing. Other statistical graphs were generated using our self-developed R codes.

## Supplementary Material

Supplementary File

## Data Availability

All study data are included in the article and *SI Appendix*.
