# Supplementary material for: Analysis of genomic distributions of SARS-CoV-2 reveals a dominant strain type with strong allelic associations
Source: Proc Natl Acad Sci U S A. 2020 Nov 12;117(48):30679–86. doi: 10.1073/pnas.2007840117 (PMC7720151; doi:10.1073/pnas.2007840117)
Supplement: Supplementary File [file pnas.2007840117.sapp.pdf]

Supplemental Information

**Contents**	....	1
**Table S1**	Annotation of the signature and sub-type SNVs	2
**Table S2**	Number and proportion of the SARS-CoV-2 strains in our defined six types	3
**Fig. S1**	Comparison of five phylogenetic and hierarchical clustering dendrograms with their defining types for 1,932 SARS-CoV-2 strains	4
**Fig. S2**	Construction of the UPGMA dendrogram for Kimura-2 distance matrix and complete-linkage dendrogram for simple matching similarity matrix with the mechanism of defining types	5
**Fig. S3**	Maximum parsimony dendrogram, variation matrix map and defined six types for 1,932 SARS-CoV-2 viral strains	6
**Fig. S4**	Temporal distributions of the six strain types in the other nations that contributed significant sample sizes	7
**Fig. S5**	Temporal pattern of pairwise allelic association for the Type VI signature SNVs C3037T, C14408T, A23403G and their haplotype frequencies	22
**Fig. S6**	Temporal trends of the occurrence counts for three, two, and one of the Type VI signature SNVs	23
**Fig. S7**	Occurrence counts of three, two, and one of Type VI signature SNVs	24
**Fig. S8**	Number of variation gain and loss in the Type VI strains in the nations that contributed a significant sample size	25
**Fig. S9**	Temporal variation frequency of the signature and sub-type SNVs	31
**Fig. S10**	Proportions of the Type VI strains in countries	32

www.pnas.org/cgi/doi/10.1073/pnas.2007840117

Table S1. Annotation of the signature and sub-type SNVs.

*Nucleotide (Gene)	**Base Change	Annotation Type	Protein:Position	Amino Acids Change	Codon Number: Codon Change	Alternate Allele Frequency (n = 1,932)	Alternate Allele Frequency (n = 6,228)	Type of Strain
nt241 (5'UTR)	C -> T	upstream gene variant	QHD43415.1	-	-	0.43	0.60	VI
nt1059 (nsp2)*	C -> T	missense variant	QHD43415.1:p.265	T>I	c.794:aCc>aTc	0.11	0.21	Sub-VIa
nt1397 (nsp2)	G -> A	missense variant	QHD43415.1:p.378	V>I	c.1132Gta>Ata	0.03	0.02	V
nt3037 (nsp3)	C -> T	synonymous variant	QHD43415.1:p.924	F	c.2772:ttC>ttT	0.43	0.60	VI
nt8782 (nsp4)	C -> T	synonymous variant	QHD43415.1:p.2839	S	c.8517:agC>agT	0.25	0.17	II, IV
nt11083 (nsp6)	G -> T	missense variant	QHD43415.1:p.3606	L>F	c.10818:ttG>ttT	0.14	0.13	III, V
nt14408 (nsp12)	C -> T	missense variant	QHD43415.1:p.4715	P>L	c.14144:cCt>cTt	0.43	0.60	VI
nt14805 (nsp12)	C -> T	synonymous variant	QHD43415.1:p.4847	Y	c.14541taC>taT	0.07	0.10	III
nt17747 (nsp13)	C -> T	missense variant	QHD43415.1:p.5828	P>L	c.17483:cCt>cTt	0.18	0.10	IV
nt17858 (nsp13)	A -> G	missense variant	QHD43415.1:p.5865	Y>C	c.17594:tAt>tGt	0.18	0.11	IV
nt18060 (nsp14)	C -> T	synonymous variant	QHD43415.1:p.5932	L	c.17796:ctC>ctT	0.18	0.11	IV
nt23403 (S)	A -> G	missense variant	QHD43416.1:p.614	D>G	c.1841:gAt>gGt	0.44	0.61	VI
nt25563 (ORF3a)*	G -> T;	missense variant	QHD43417.1:p.57	Q>H	c.171:caG>caT	0.14	0.25	Sub-VIa
nt26144 (ORF3a)	G -> T	missense variant	QHD43417.1:p.251	G>V	c.752gGt>gTt	0.08	0.10	III
nt28144 (ORF8)	T -> C	missense variant	QHD43422.1:p.84	L>S	c.251:tTa>tCa	0.25	0.17	II, IV
nt28688 (N)	T -> C	synonymous variant	QHD43423.2:p.139	L	c.415Ttg>Ctg	0.03	0.02	V
nt28881 (N)*	G -> A	missense variant	QHD43423.2:p.203	R>K	c.608:aGg>aAg	0.13	0.15	Sub-VIb
nt28882 (N)*	G -> A	synonymous variant	QHD43423.2:p.203	R>K	c.609:agG>aaA	0.13	0.15	Sub-VIb
nt28883 (N)*	G -> C	missense variant	QHD43423.2:p.204	G>R	c.610:Gga>Cga	0.13	0.15	Sub-VIb

*Sub-type SNVs. ** Only list the major nucleotide if there are more than one variation.

Table S2. Number and proportion of the SARS-CoV-2 strains in our defined six types. Strain types were defined based on an analysis of 1,932 SARS-CoV-2 genomes and validated in two subsequent analyses of 6,228 and 38,248 SARS-CoV-2 genomes. In the first data set (n = 1,932, downloaded on March 31, 2020), the six types of the SARS-CoV-2 strains (Type I – VI) were defined according to the maximum parsimony dendrogram. Then, 13 signature SNVs was applied to assign strain types for all SARS-CoV-2 strains in the second data set (n = 6,228, downloaded on April 19, 2020). Three methods of strain typing by using the 13 signature SNVs were applied: (a) Based on the sequence data processed with a time-consuming multiple sequence alignment; (b) Based on the sequence data processed with a pairwise alignment (without the need for multiple sequence alignment); (c) Based on a text mining approach (without the need for multiple sequence alignment). In the third data set (n = 38,248, downloaded on June 8, 2020), a multiple sequence alignment for this large data set was not performed because of computational expense. Only two methods of strain typing by using the 13 signature SNVs were applied: (a) Based on the sequence data processed with a pairwise alignment; (b) Based on a text mining approach. In each of the aforementioned analyses, computational time and proportions of the six strain types are shown.

Dataset	Method	Time	I	II	III	IV	V	VI	Other
n = 1,932	Maximum parsimony tree	3,617 min	428 (22.1%)	150 (7.8%)	114 (5.9%)	341 (17.7%)	53 (2.7%)	844 (43.7%)	2 (0.2%)
n = 6,228	Signature SNVs & multiple sequence alignment	21,273 min	756 (12.1%)	384 (6.2%)	511 (8.2%)	645 (10.4%)	103 (1.7%)	3745 (60.1%)	84 (1.3%)
	Signatures SNVs & pairwise alignment	360 min	756 (12.1%)	384 (6.2%)	511 (8.2%)	645 (10.4%)	103 (1.7%)	3745 (60.1%)	84 (1.3%)
	Signatures SNVs & our text mining algorithm	0.85 min	756 (12.1%)	384 (6.2%)	501 (8%)	645 (10.4%)	103 (1.7%)	3745 (60.1%)	94 (1.5%)
n = 38,248	Signatures SNVs & pairwise alignment	2,212 min	3791 (9.9%)	1644 (4.3%)	2872 (7.5%)	1388 (3.6%)	262 (0.7%)	27887 (72.9%)	404 (1.1%)
	Signatures SNVs & our text mining algorithm	5.48 min	3890 (10.2%)	1595 (4.2%)	2839 (7.4%)	1376 (3.6%)	257 (0.7%)	27747 (72.5%)	544 (1.4%)

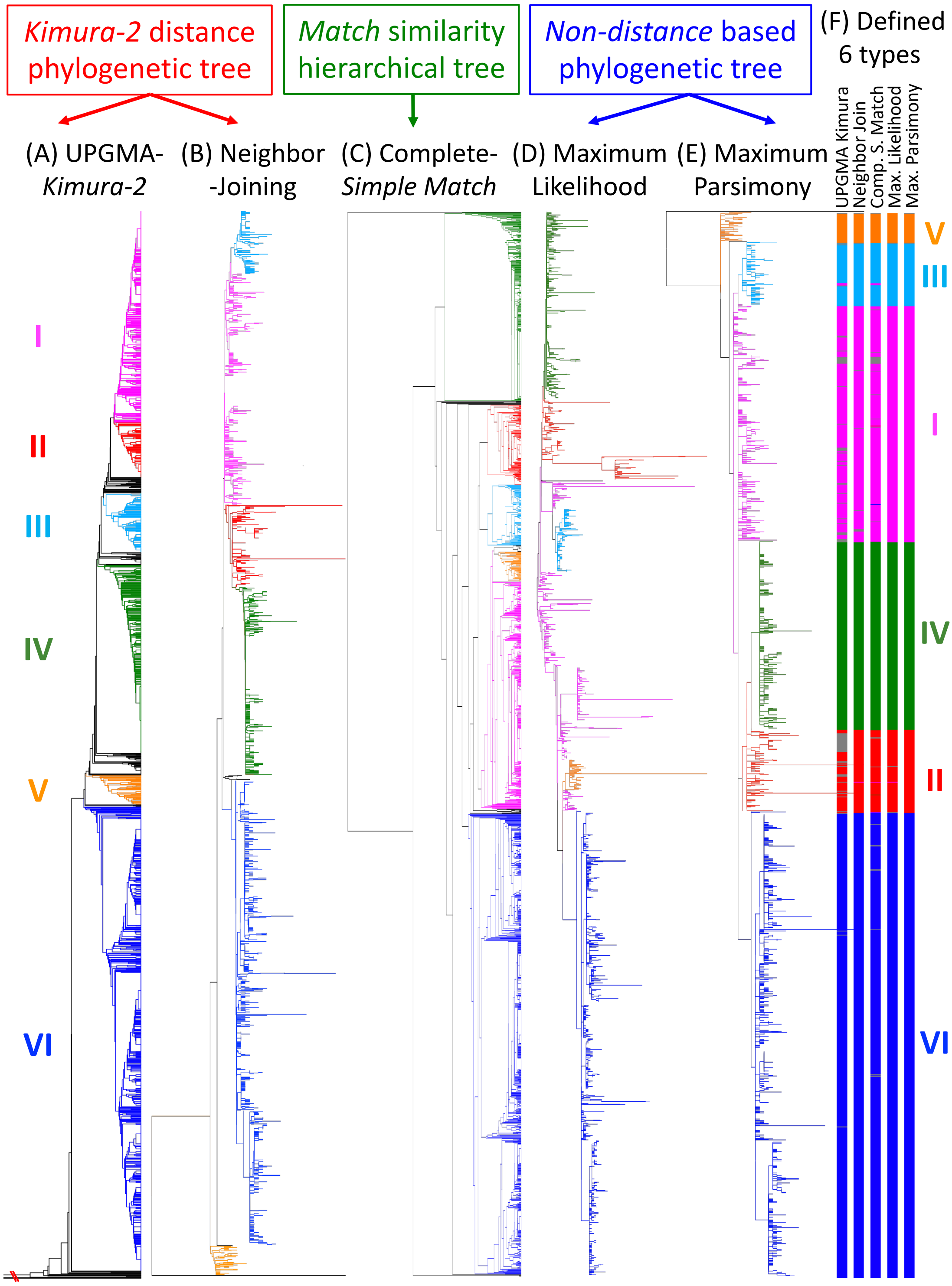

Fig. S1. Comparison of five phylogenetic and hierarchical clustering dendrograms with their defining types for 1,932 SARS-CoV-2 strains. Five dendrograms are built for 1,932 SARS-CoV-2 strains with each strain in every dendrogram represented by one terminal node (leave). A binary variation matrix for 2,139 variation sites with at least one variation among all 1,932 strains is utilized to assist each dendrogram in identifying strain types for that dendrogram. (A) UPGMA (unweighted pair group method with arithmetic mean) phylogenetic tree built on Kimura 2-parameter distance matrix computed from whole genome information with 29,903 nucleotides (type IDs and colors used by this dendrogram are adopted by the rest four dendrograms); (B) Neighbor-Joining phylogenetic tree built on Kimura 2-parameter distance matrix; (C) Complete-linkage hierarchical clustering tree built on simple matching similarity (equivalent to (1 – City-block distance)) matrix computed from 1,932 strains with 2,139 variation sites; (D) Maximum likelihood phylogenetic tree built on whole genome information with 29,903 nucleotides; (E) Maximum parsimony phylogenetic tree built on whole genome information with 29,903 nucleotides; (F) Color panels for defined types by five dendrograms with 1,932 strains sorted by terminal node (leaves) order from the maximum parsimony dendrogram in (E). Apparently all 5 dendrograms assigned very similar type memberships for all 1,932 strains with only minor discrepancies. Figure S2 has more detailed descriptions on the construction of the UPGMA dendrogram for Kimura 2-parameter distance matrix and the complete-linkage dendrogram on the simple matching similarity matrix with the mechanism of defining types.

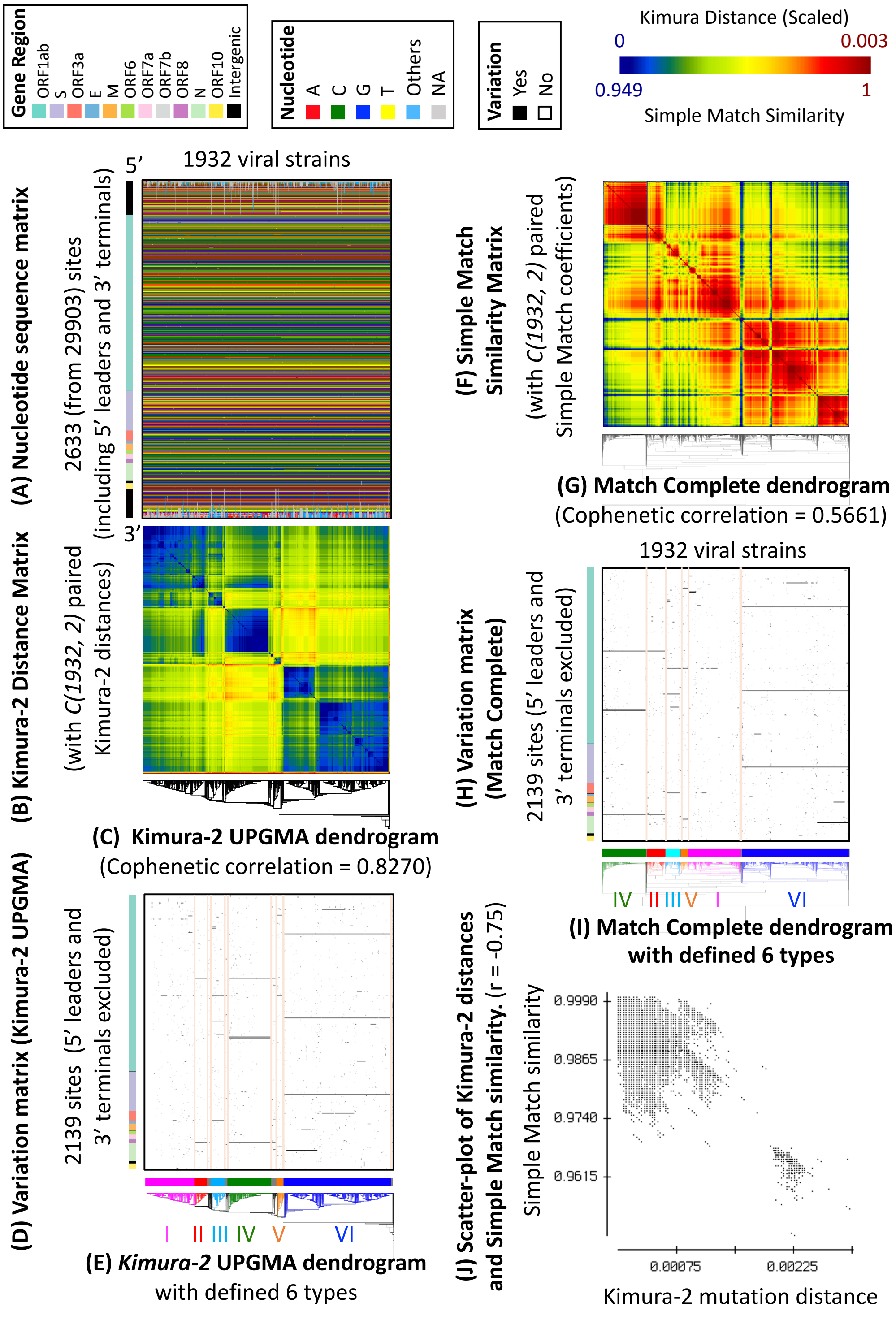

Fig. S2. Construction of the UPGMA dendrogram for Kimura-2 distance matrix and complete-linkage dendrogram for simple matching similarity matrix with the mechanism of defining types. (A) Whole genome nucleotide sequence matrix for 1,932 viral strains with 29,903 SNP sites (only 2,633 sites with variations are displayed); (B) Kimura-2 distance matrix map with *C(1,932, 2)* paired Kimura 2-paremeter distances computed from (A); (C) UPGMA dendrogram built on Kimura-2 distance matrix with Cophenetic correlation equals 0.827; (D) Variation matrix map for 2,139 variation sites on 1,932 viral strains with strains sorted by leave nodes order of Kimura-2 UPGMA dendrogram in (C); (E) Color coded Kimura-2 UPGMA dendrogram with six types defined using dendrogram branching structure in (C) and variation patterns in (D); (F) Matrix map of simple matching similarity, which is Equivalent to (1 – City-block) distance, with *C(1,932, 2)* paired simple matching coefficients computed from (D); (G) Complete linkage dendrogram built on simple matching similarity matrix (with Cophenetic correlation 0.5661); (H) Variation matrix map for 2,139 variation sites on 1,932 viral strains with strains sorted by leave nodes order of complete linkage dendrogram in (G); (I) Color coded simple match complete linkage dendrogram with six types defined using dendrogram branching structure in (G) and variation patterns in (H); (J) Scatter-plot of *C(1,932, 2)* Kimura-2 distances in (B) and simple matching similarities in (F) with correlation coefficient (Cophenetic correlation like) equals -0.75. From the scatterplot and correlation magnitude we understand the association (between the Kimura 2-parameter distance derived from the whole genome with 29,903 SNP sites and the simple matching similarity computed from only the 2,139 variation sites) is not extremely high yet the two dendrograms built from these two matrices identified nearly identical six viral types with the aid of the sorted variation matrix maps.

Fig. S3

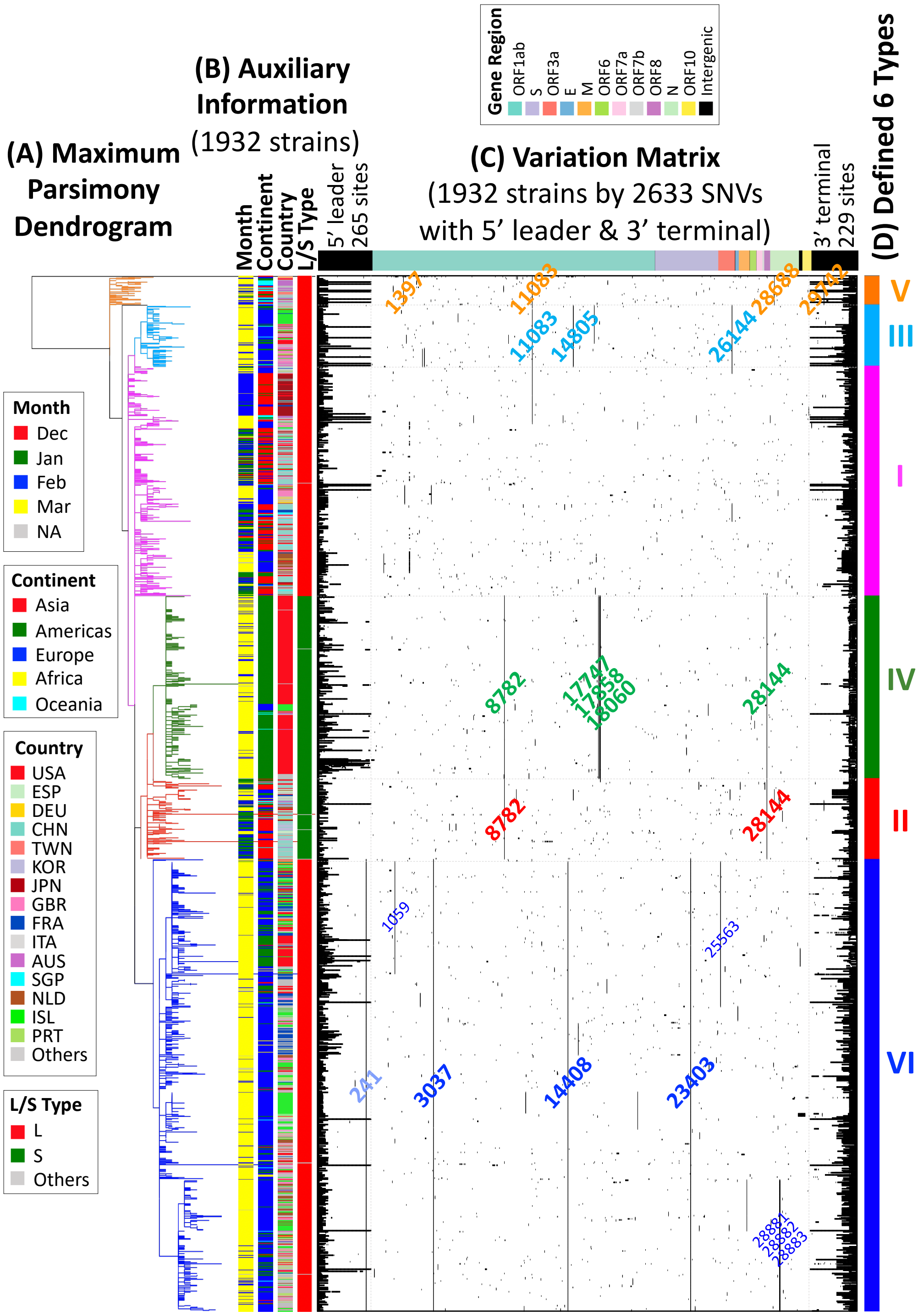

Fig. S3. Maximum parsimony dendrogram, variation matrix map and defined six types for 1,932 SARS-CoV-2 viral strains. (i) Maximum parsimony (MP) dendrogram with color coding for six types of 1,932 strains; (ii) Auxiliary information for each virus strain sorted by the (MP) dendrogram - month of data collection, continent, country, two strain types (L, S) defined by (Tang et al., 2020, National Science Review). (iii) Variation matrix map for 1,932 strains with 2,633 variation sites (including 265 sites in the 5' leader and 229 sites in the 3' terminal sequences). Strains are sorted using terminal nodes order from the MP dendrogram. Nucleotides are listed by relative positions in the genome with color bands indicating their corresponding gene regions on the top panel. A black dot at position (i, j) indicates an occurrence of a variation at nucleotide j (=1,..., 2,633) for strain i (=1,..., 1932). All 2,633 nucleotides have at least one variation among 1,932 strains. The variation structure in the sorted matrix was then combined with the dendrogram branching pattern in identifying the 6 major types with their signature SNVs. Signature variations for each type (and subtypes for Type VI) are labeled with corresponding type colors. (iv) Color bar for defined six types for 1,932 strains. Only 0.103% (2 strains) of 1,932 strains could not be assigned to the six types. From the auxiliary information panel we also observed the following facts. Strains in Types I & II were collected earlier than strains in the other types. Types II & IV correspond to the S type (Tang et al., 2020, National Science Review) with signature SNVs C8782T and T28144C, while Types I, III, V, VI correspond to the L type (Tang et al., 2020, National Science Review). Other associations among the four auxiliary covariates with the six defined types can also be identified by visualizing relative positions with colors in the auxiliary information panel and types in variation matrix map. There is one signature SNV (nt241) for type VI in the 5' leader sequences and one signature SNV (nt29742) for type V in the 3' terminal sequences respectively.

Fig. S4A.

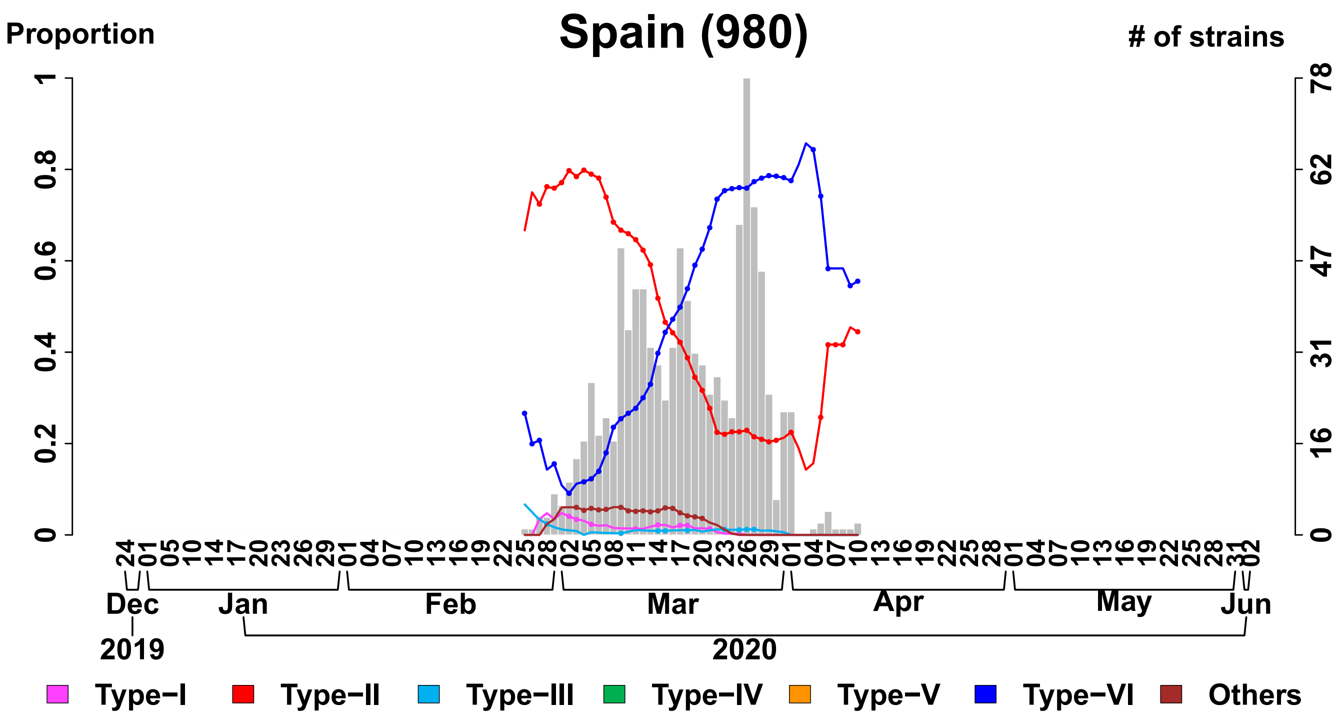

Fig. S4. Temporal distributions of the six strain types in the other nations that contributed significant sample sizes. In each subfigure, the proportions of Type I – Type VI are displayed using six curves with different colors. The left-hand-side vertical axis indicates the moving-window proportion calculated by dividing the number of the strains belonging to a specific type by the total number of the strains for the samples within four dates of a specific date in each side. The right-hand-side vertical axis indicates the number of strains (i.e., sample size). Sample size is displayed with a histogram in the background.

Fig. S4B.

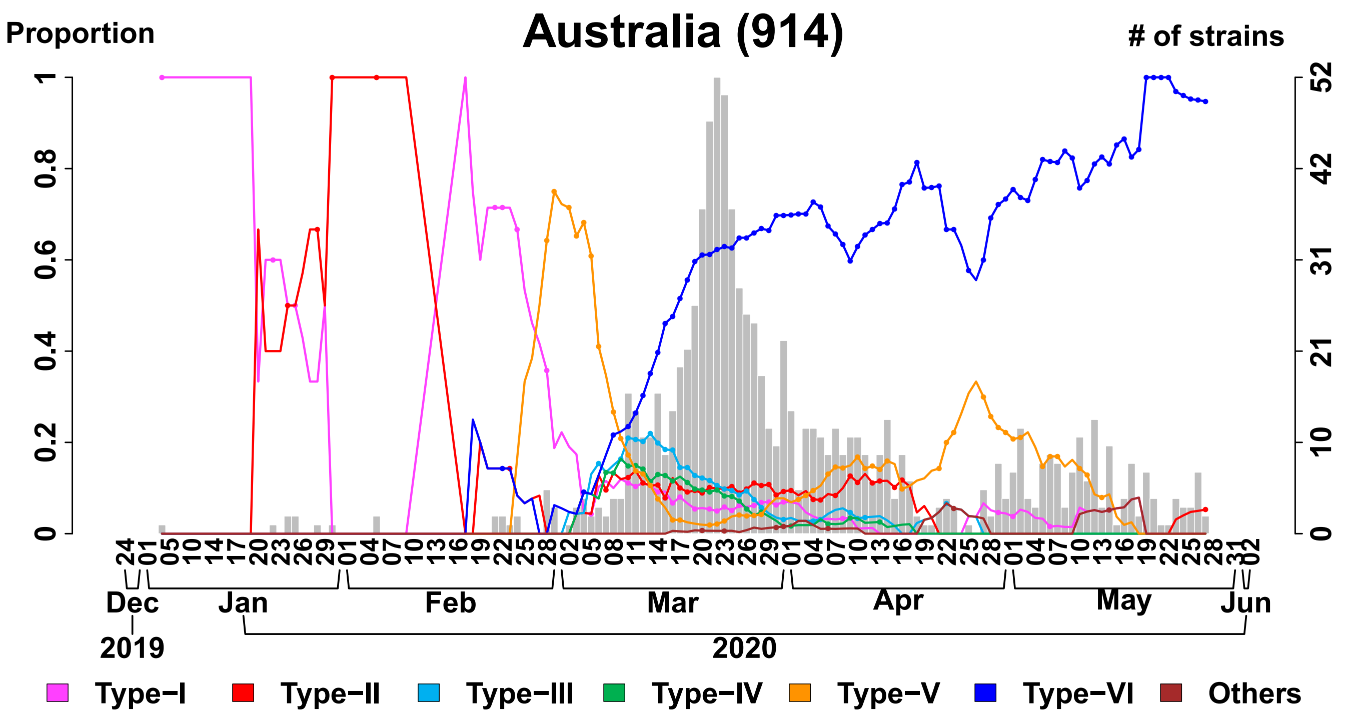

Fig. S4C.

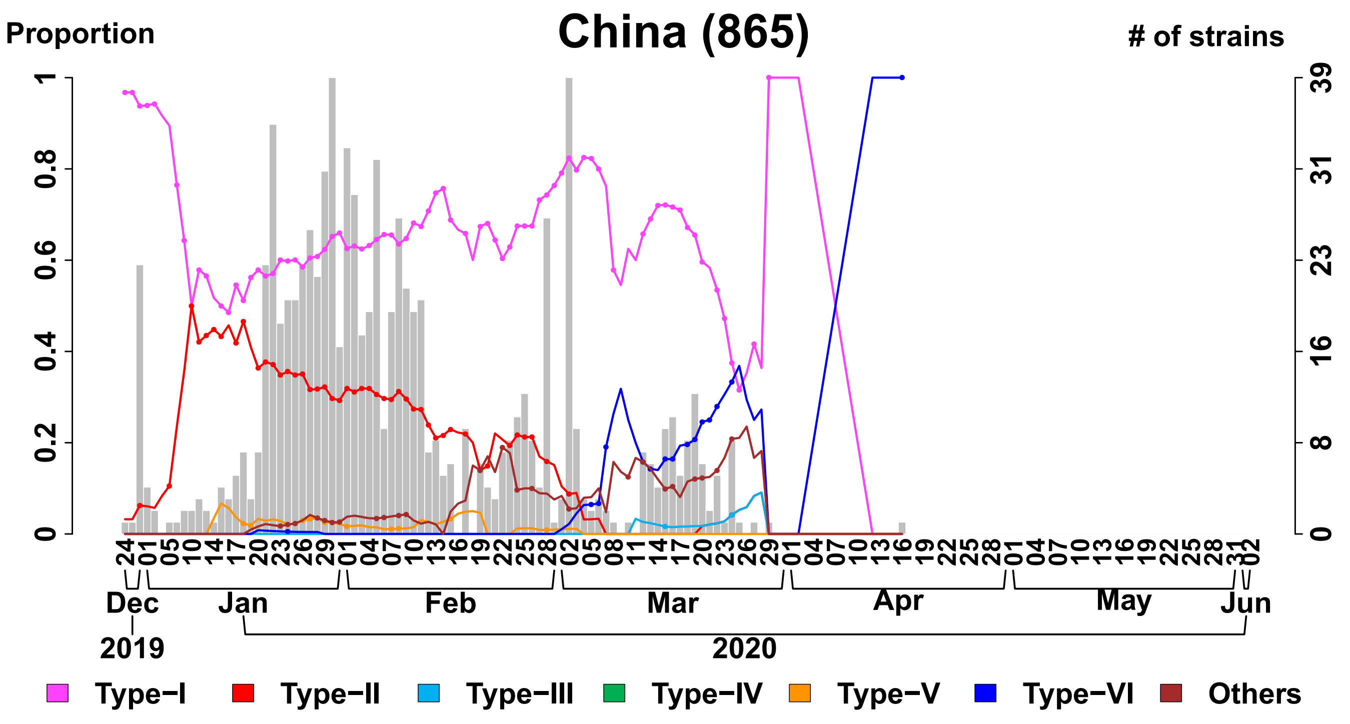

Fig. S4D.

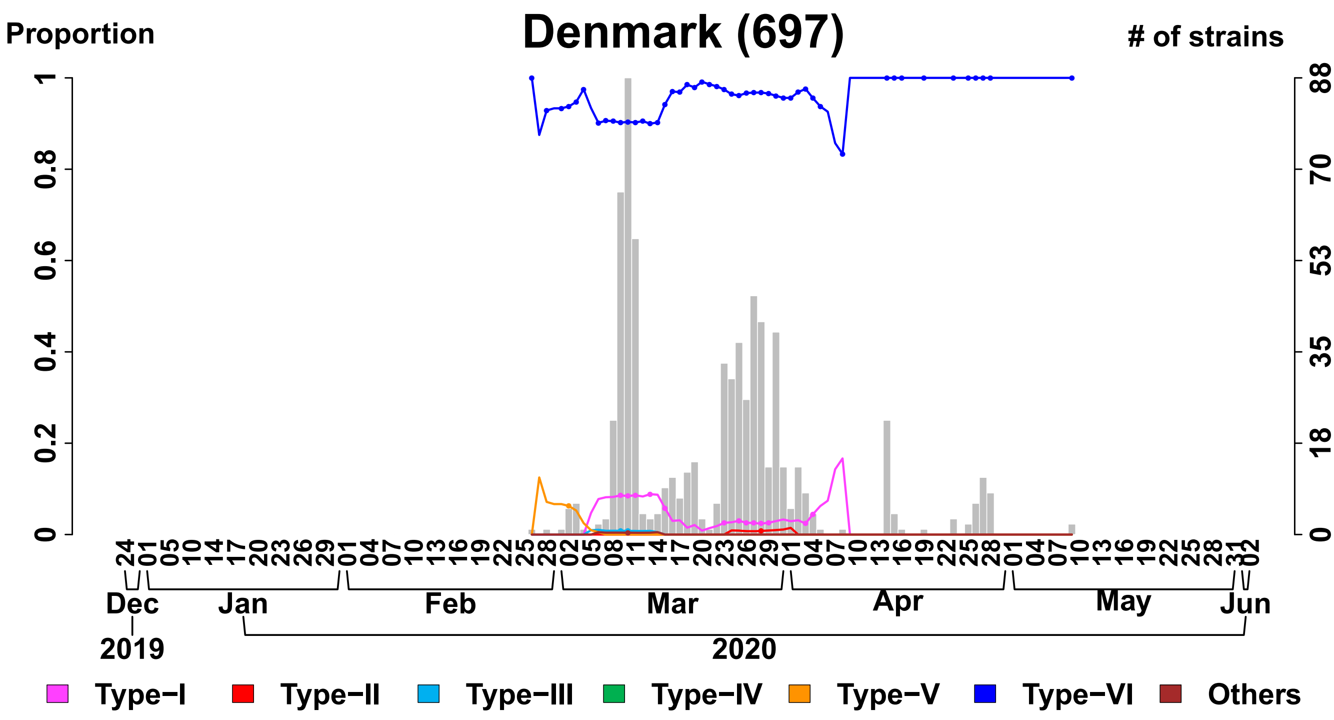

Fig. S4E.

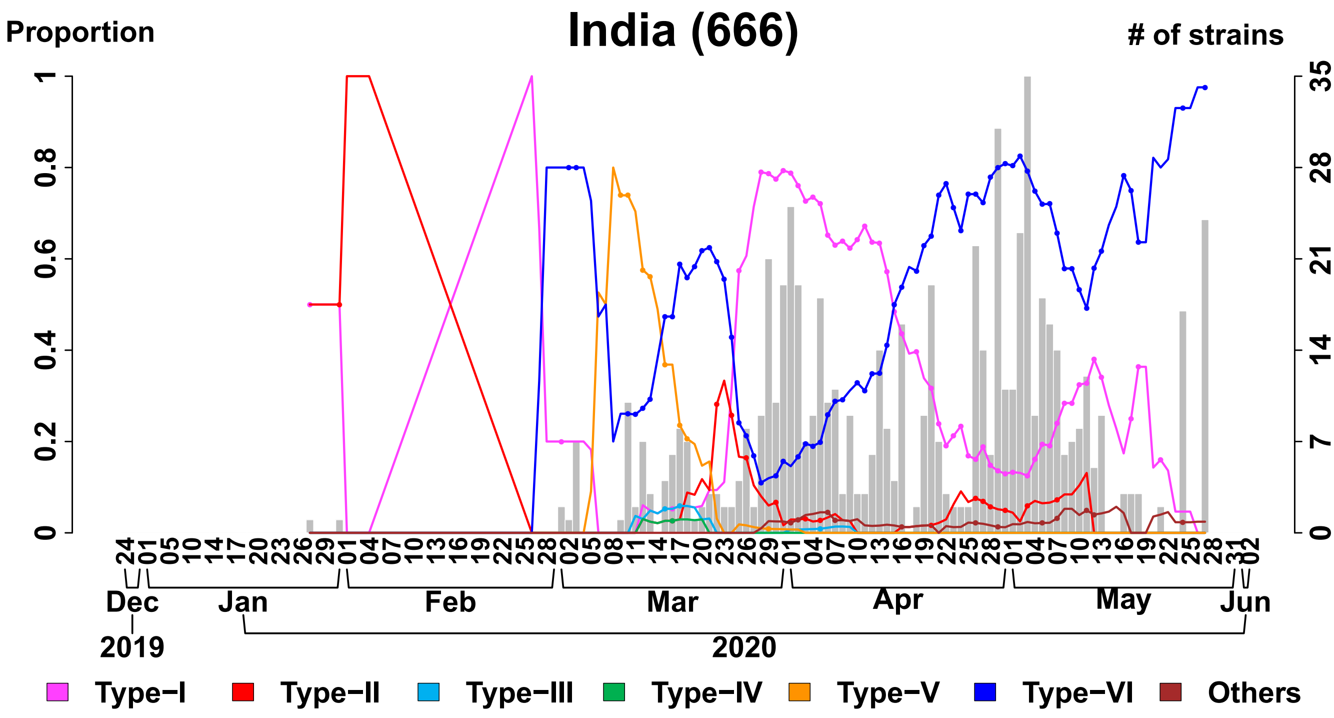

Fig. S4F.

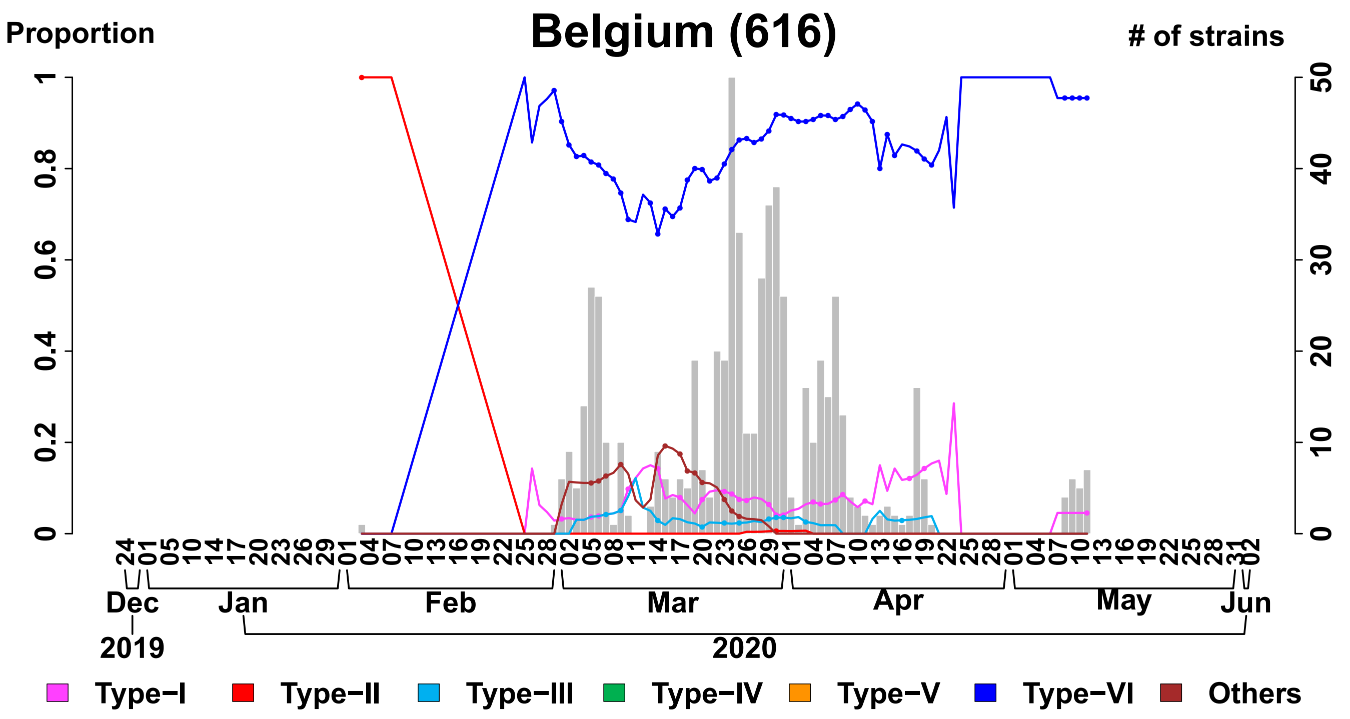

Fig. S4G.

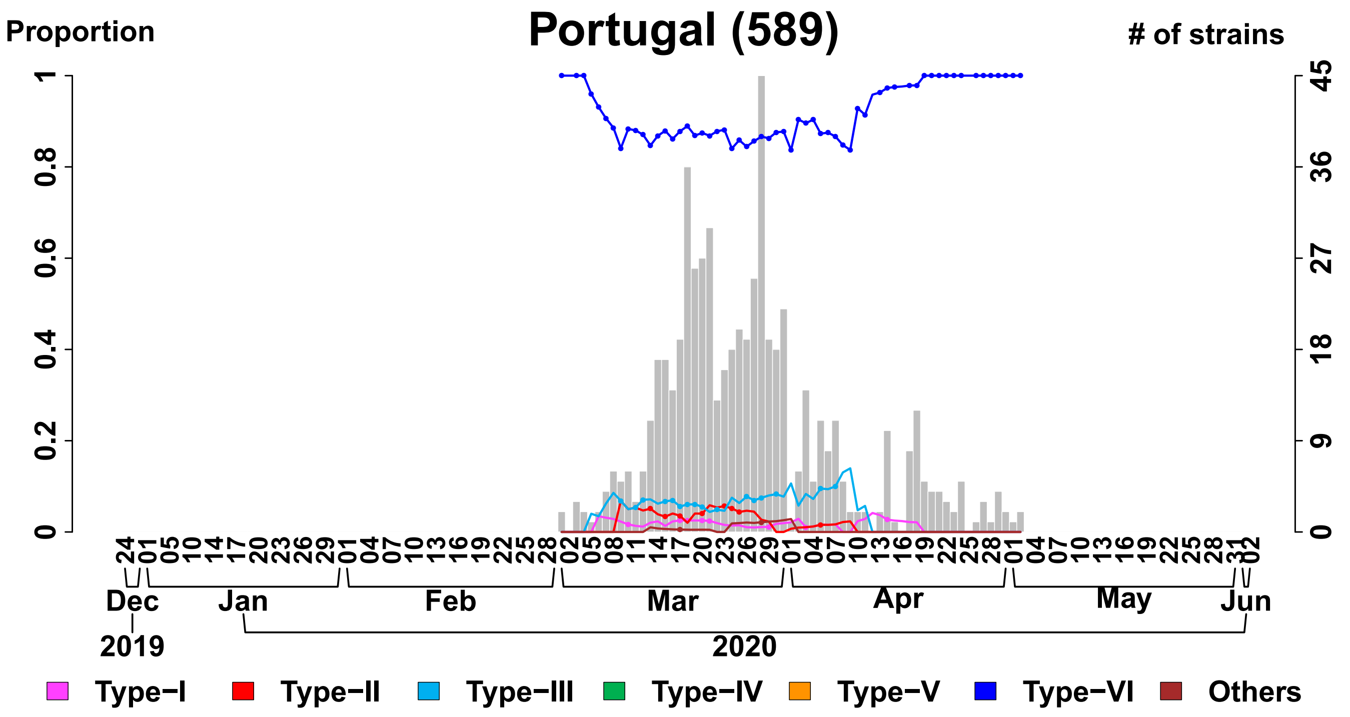

Fig. S4H.

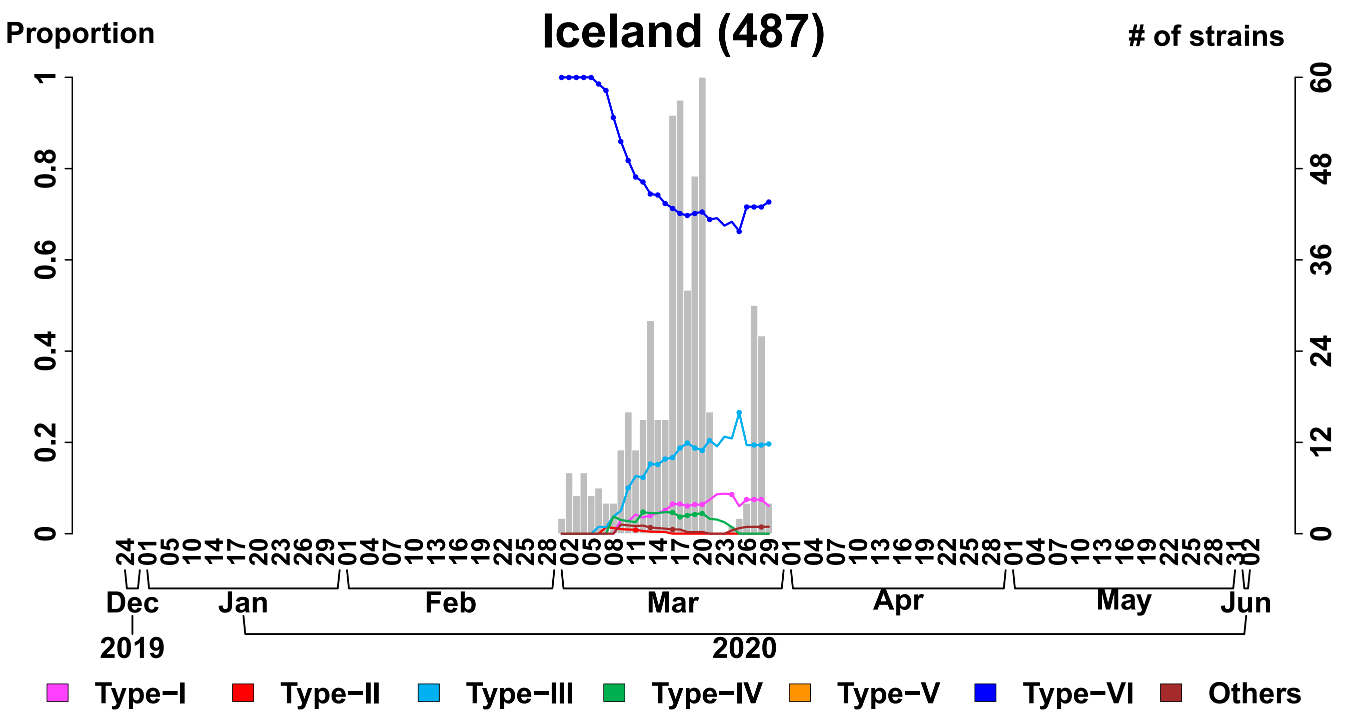

Fig. S4I.

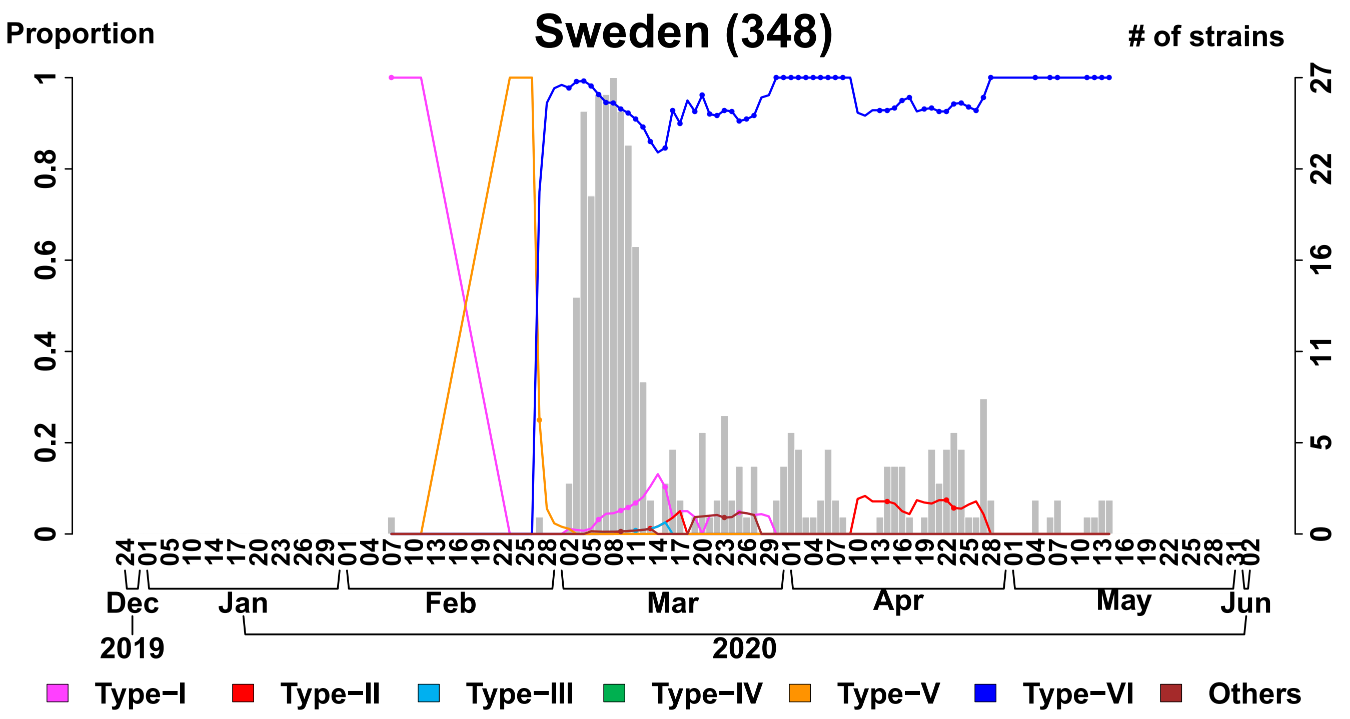

Fig. S4J.

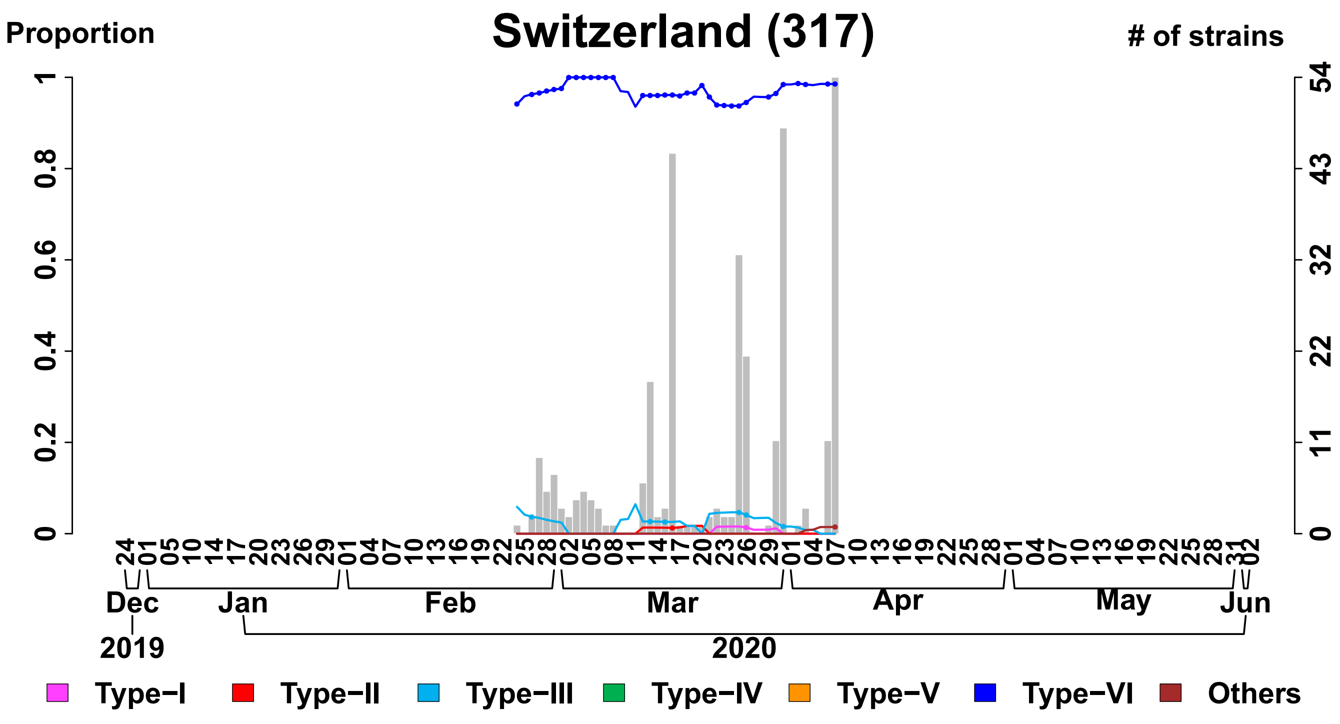

Fig. S4K.

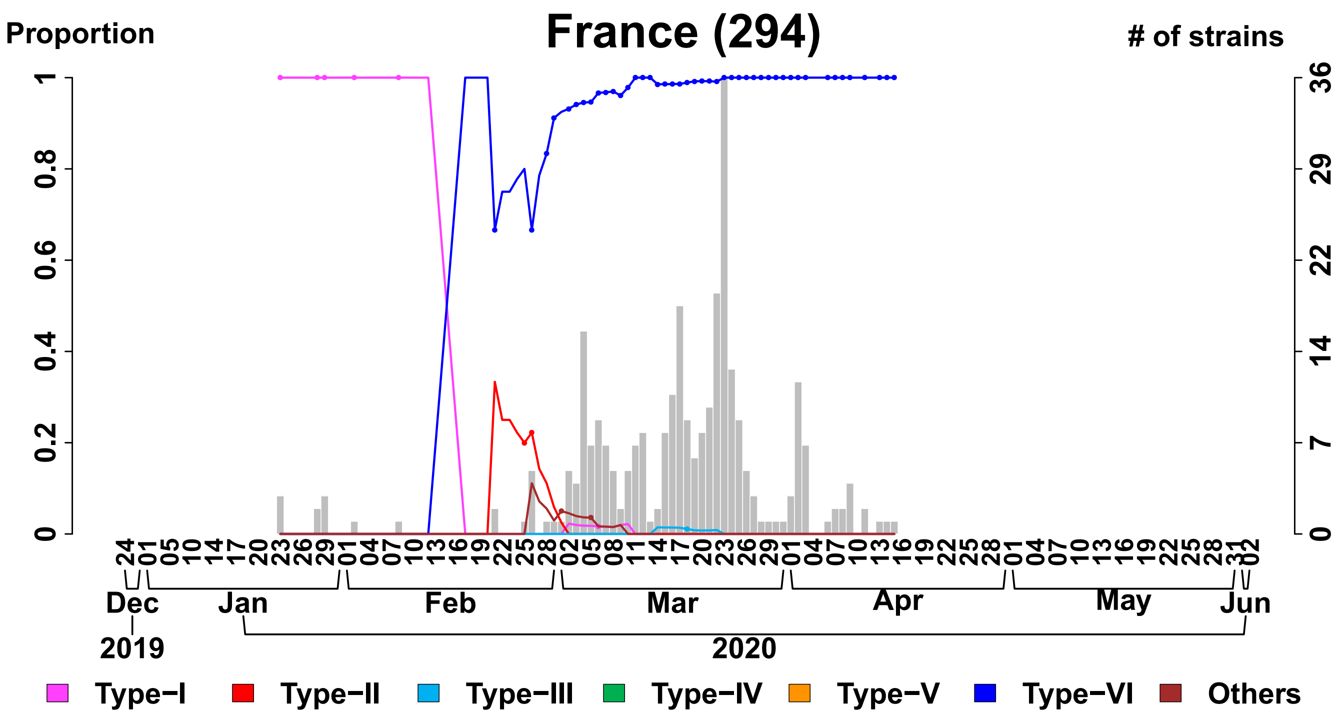

Fig. S4L.

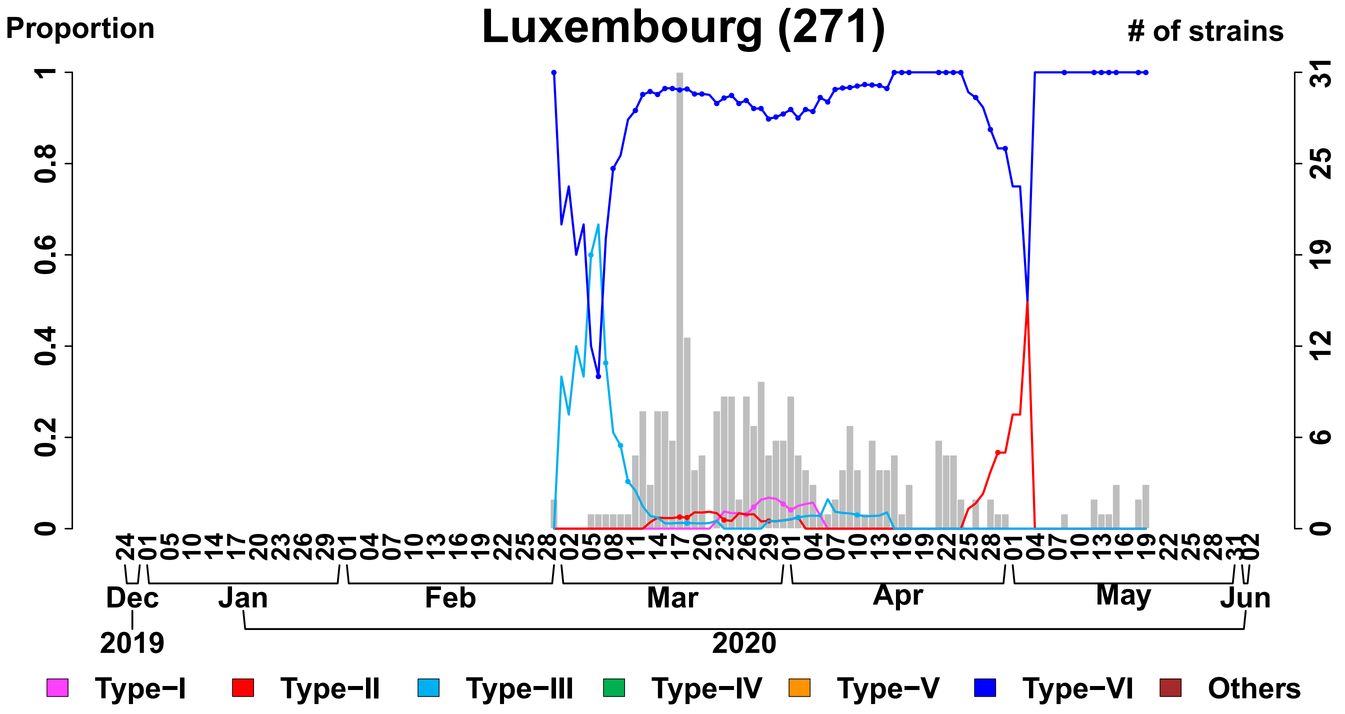

Fig. S4M.

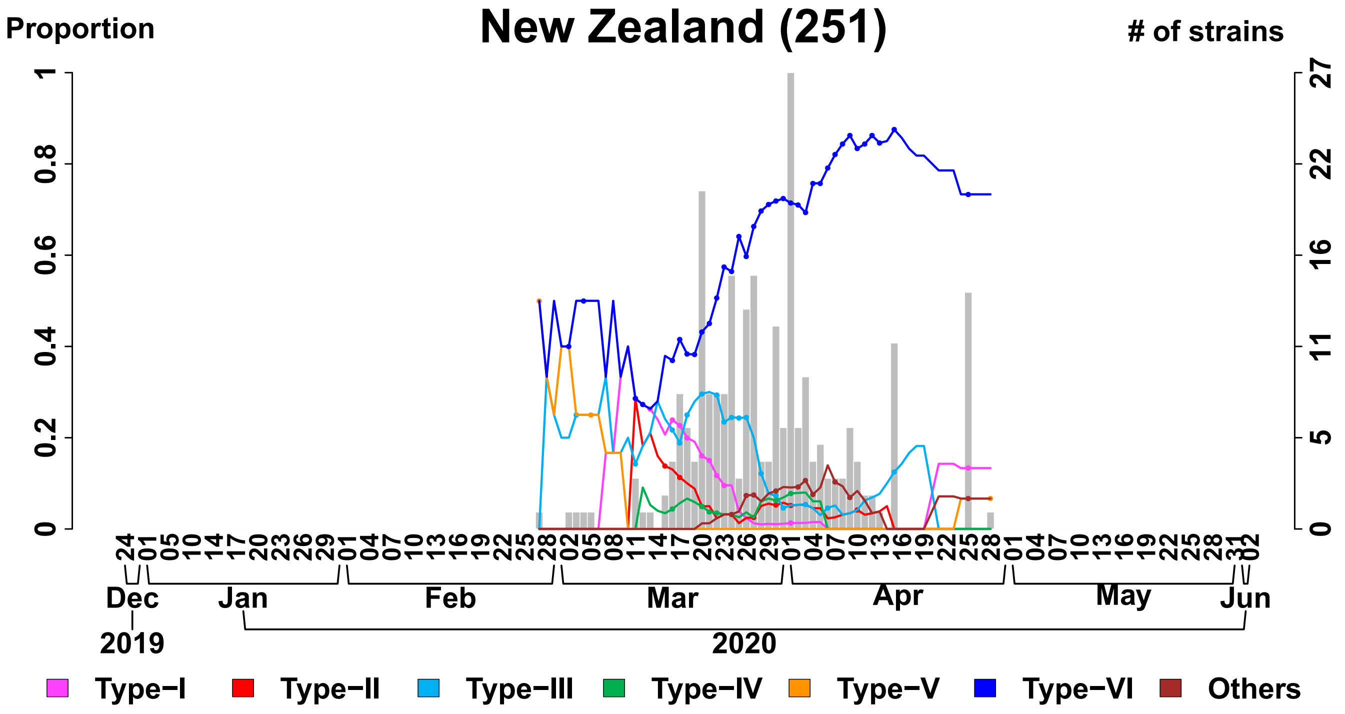

Fig. S4N.

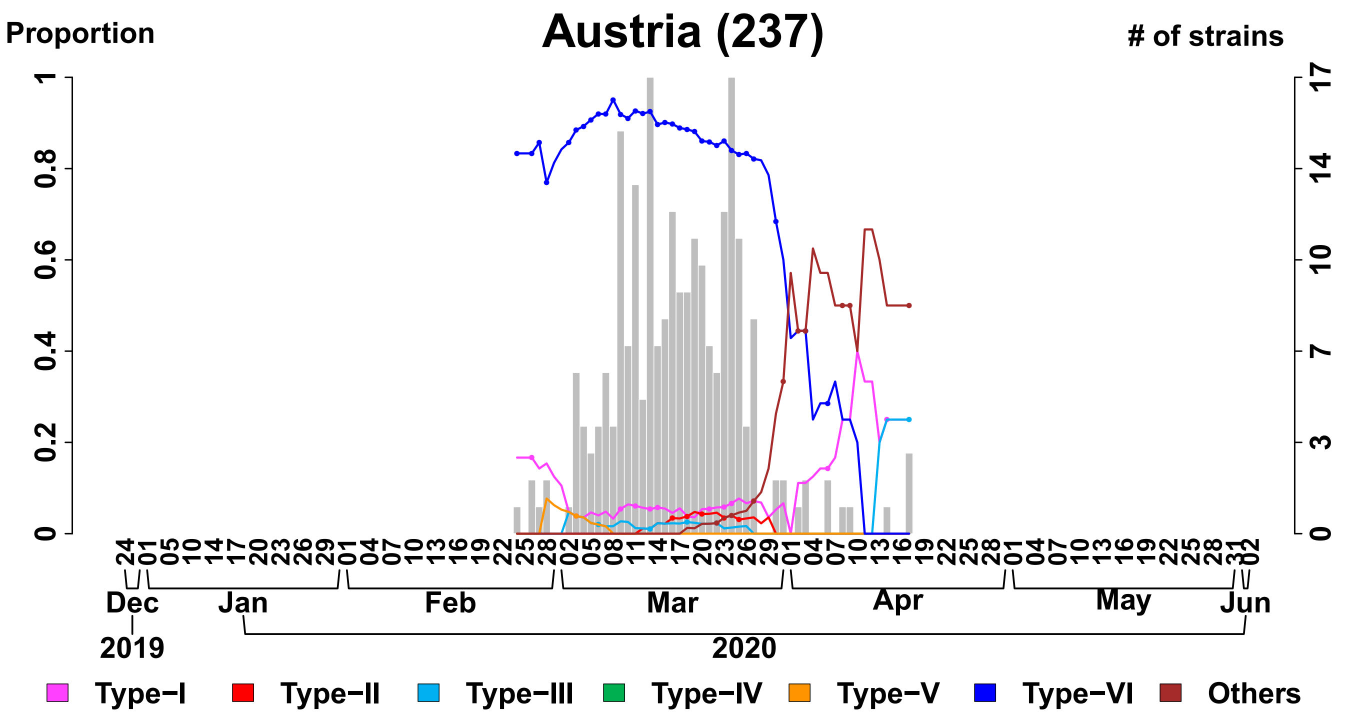

Fig. S4O.

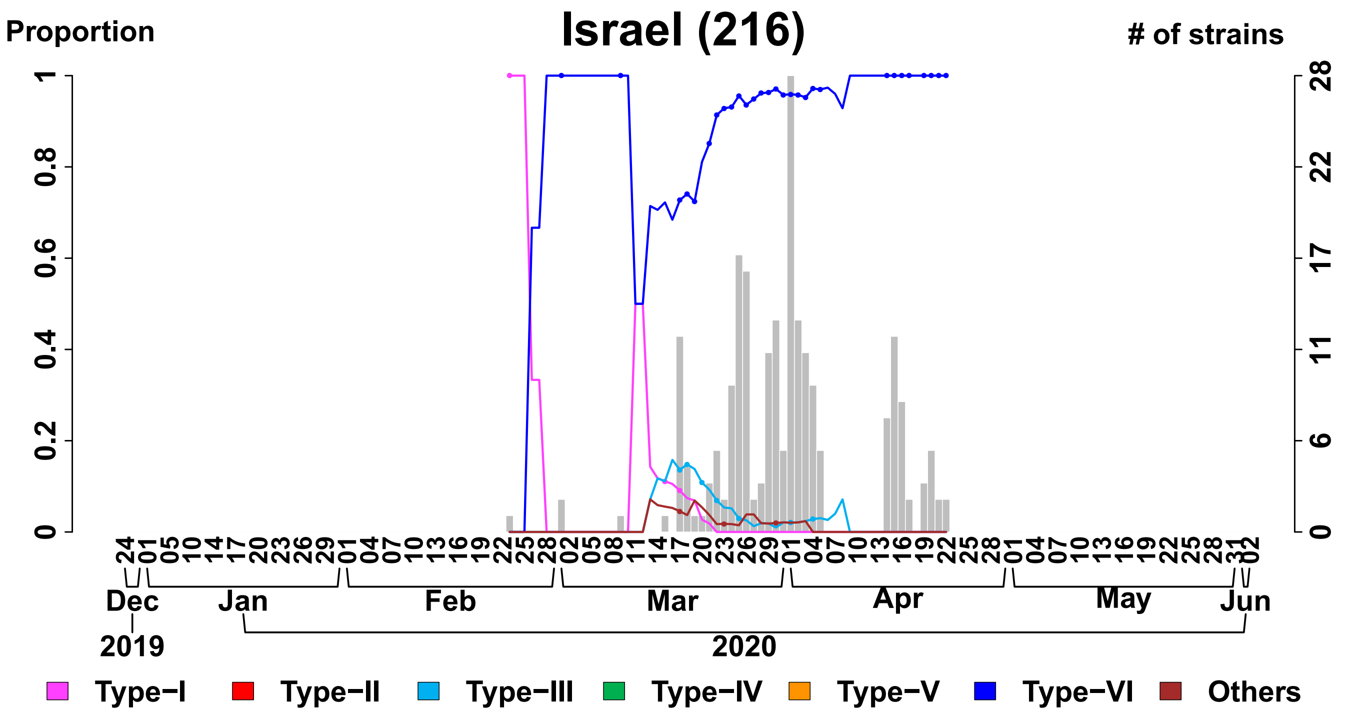

Fig. S4P.

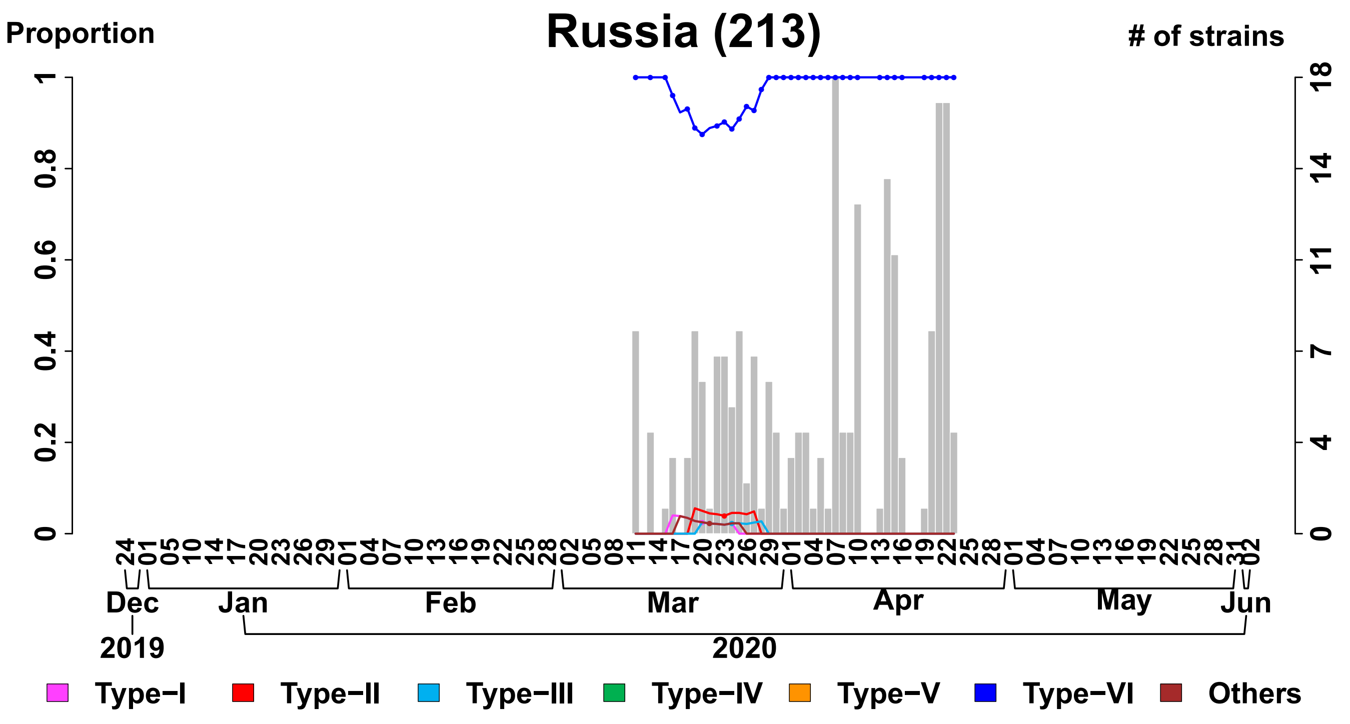

Fig. S4Q.

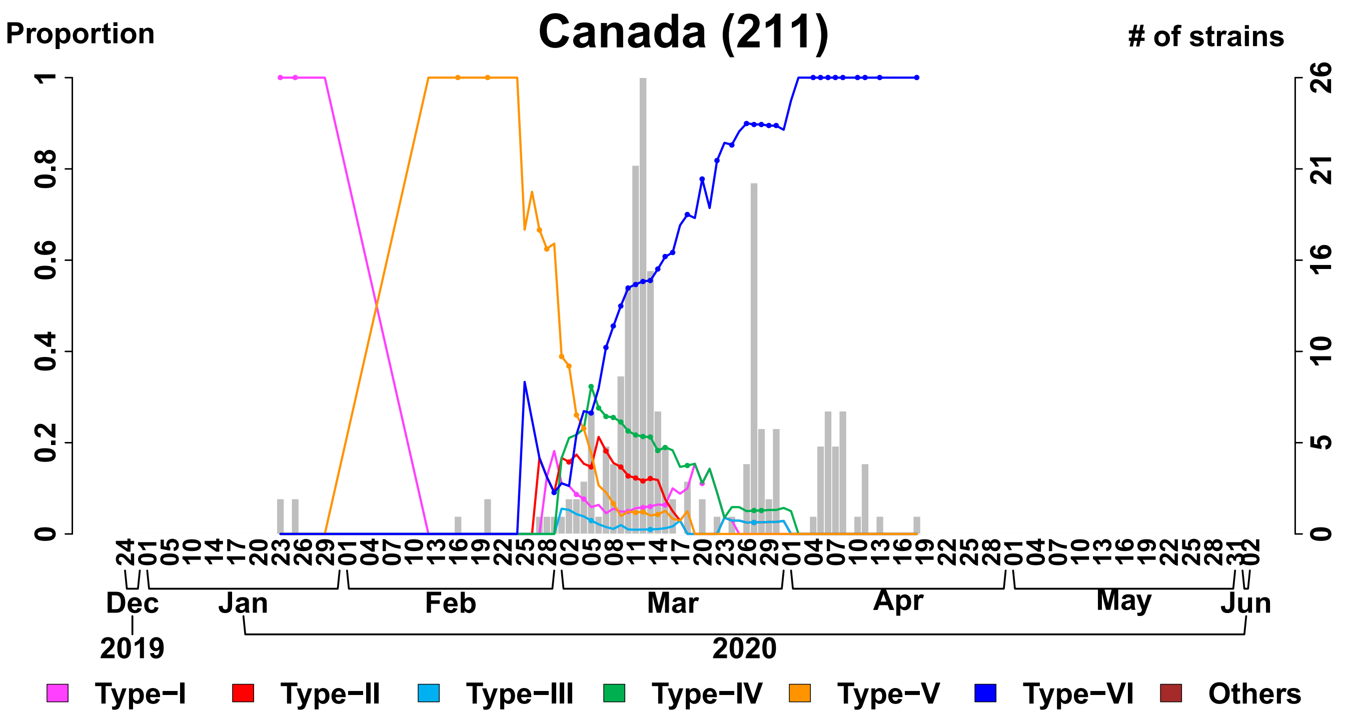

Fig. S4R.

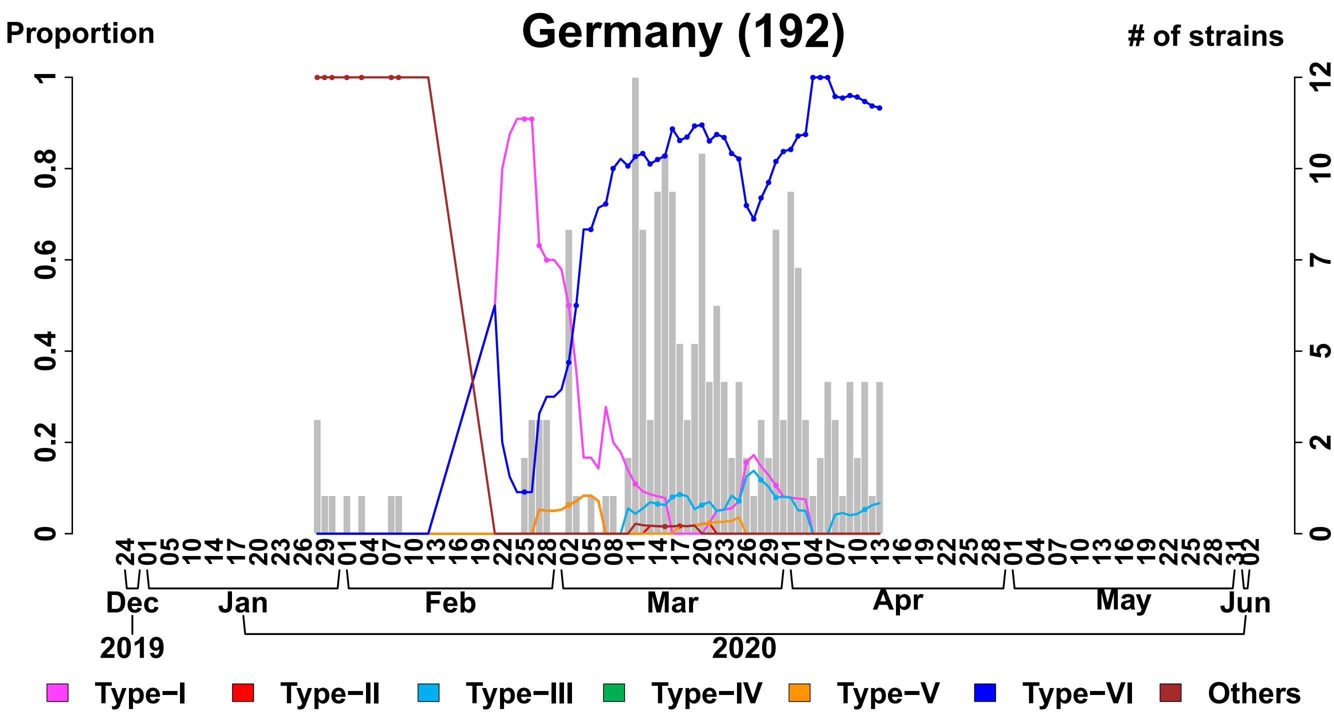

Fig. S4S.

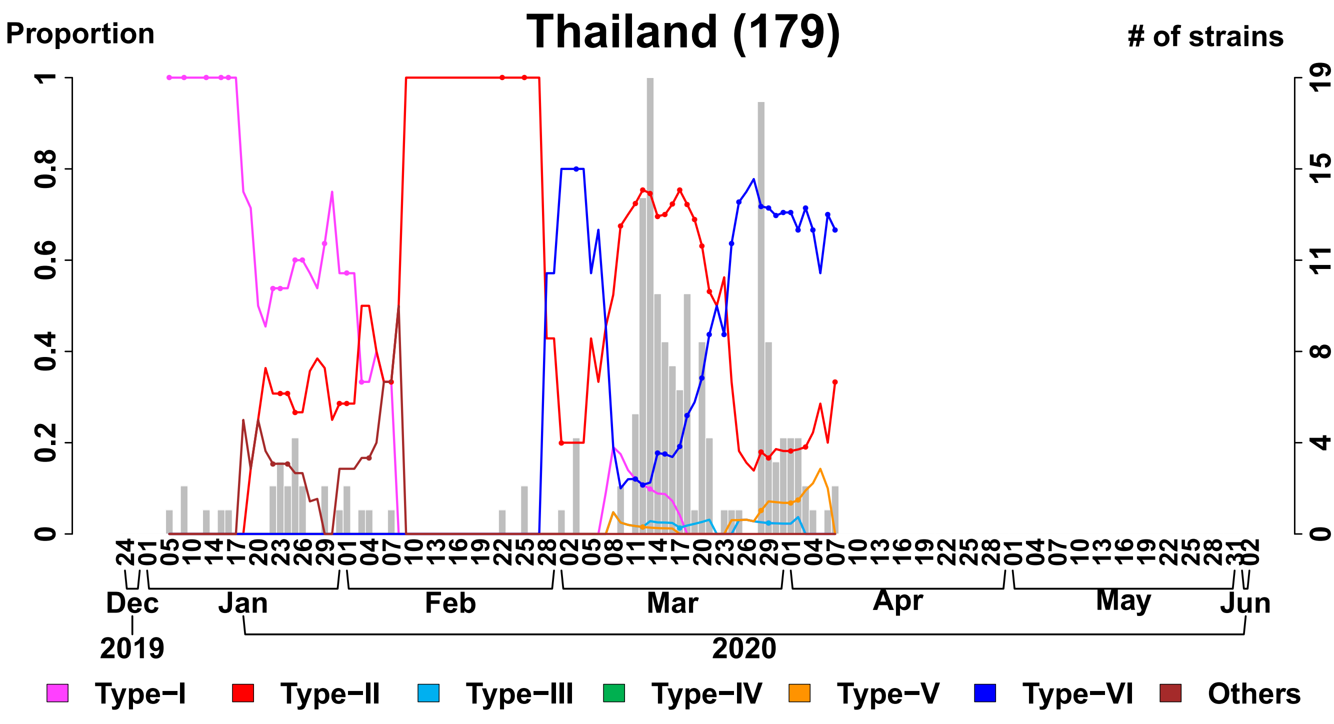

Fig. S4T.

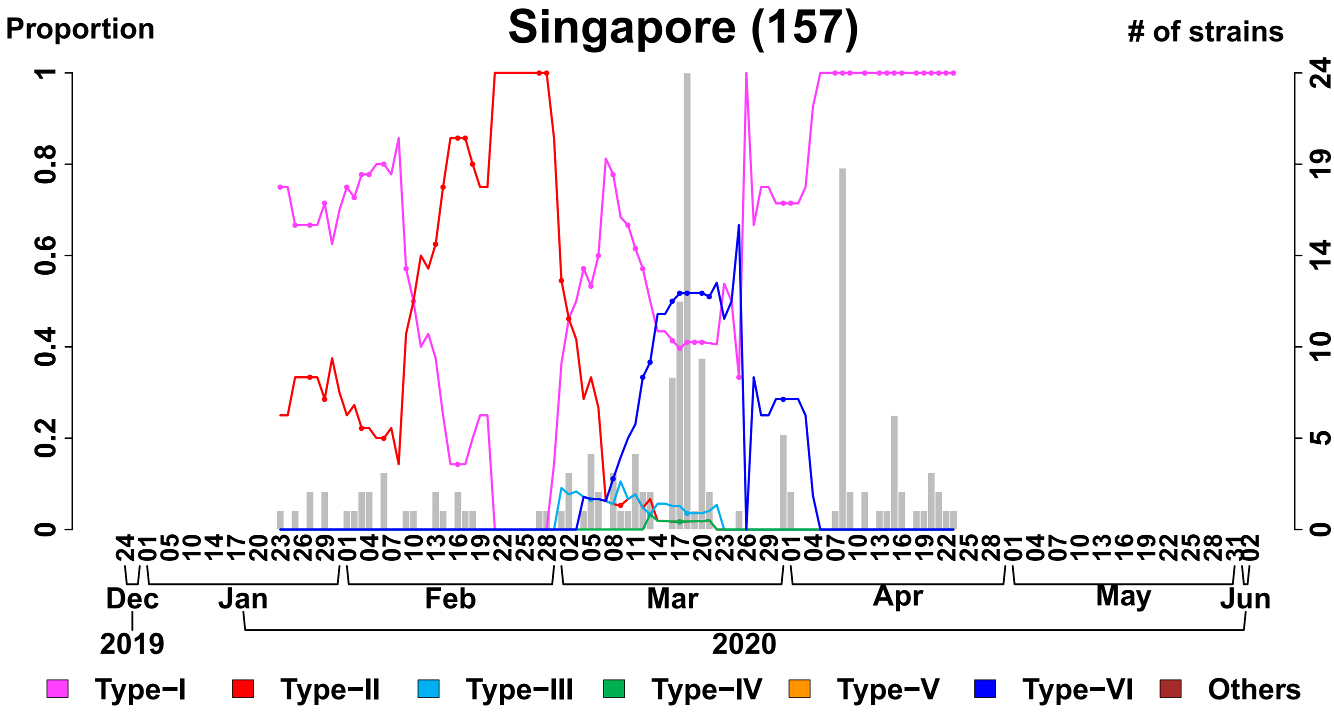

Fig. S4U.

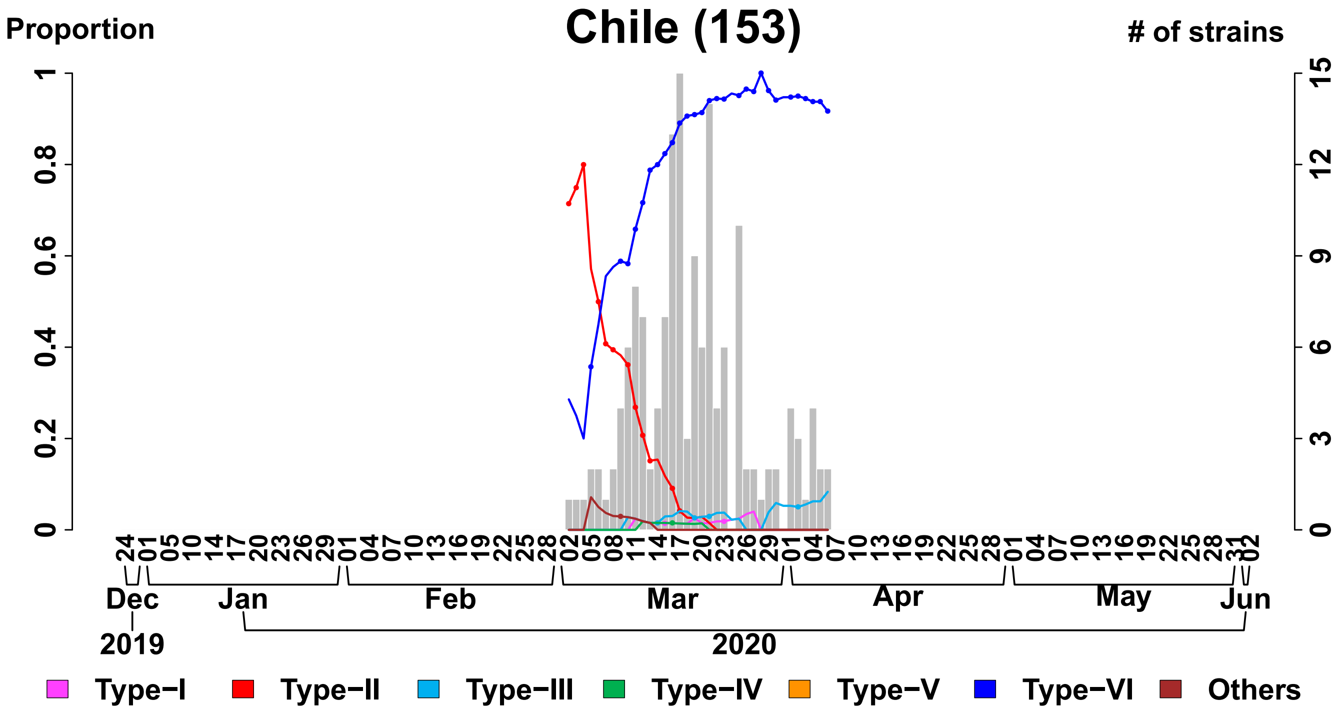

Fig. S4V.

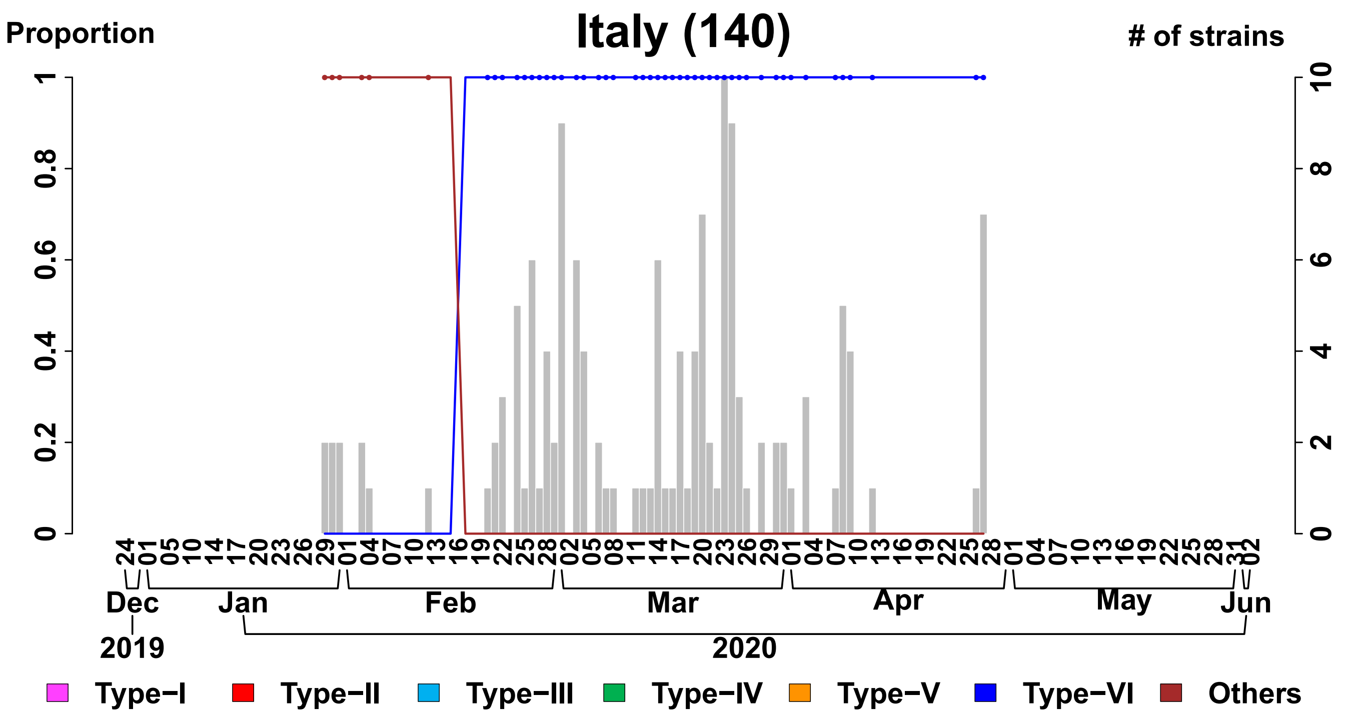

Fig. S4W.

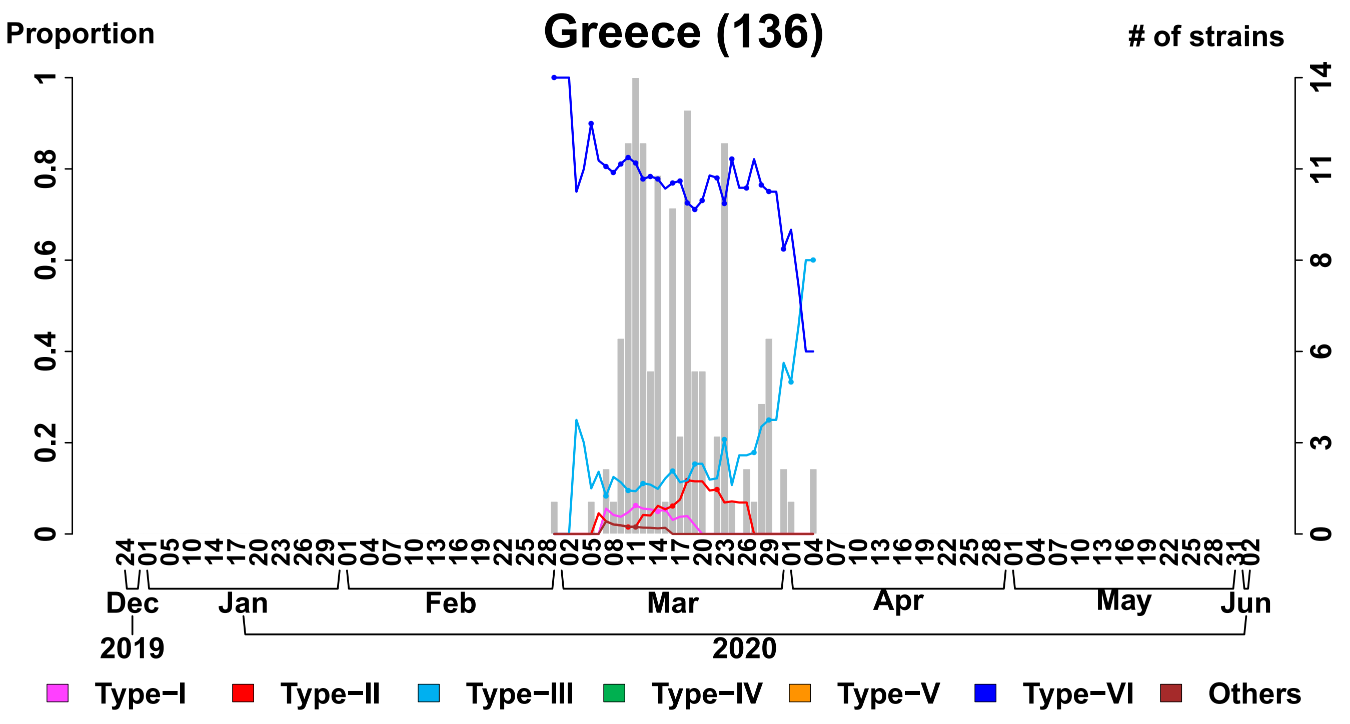

Fig. S4X.

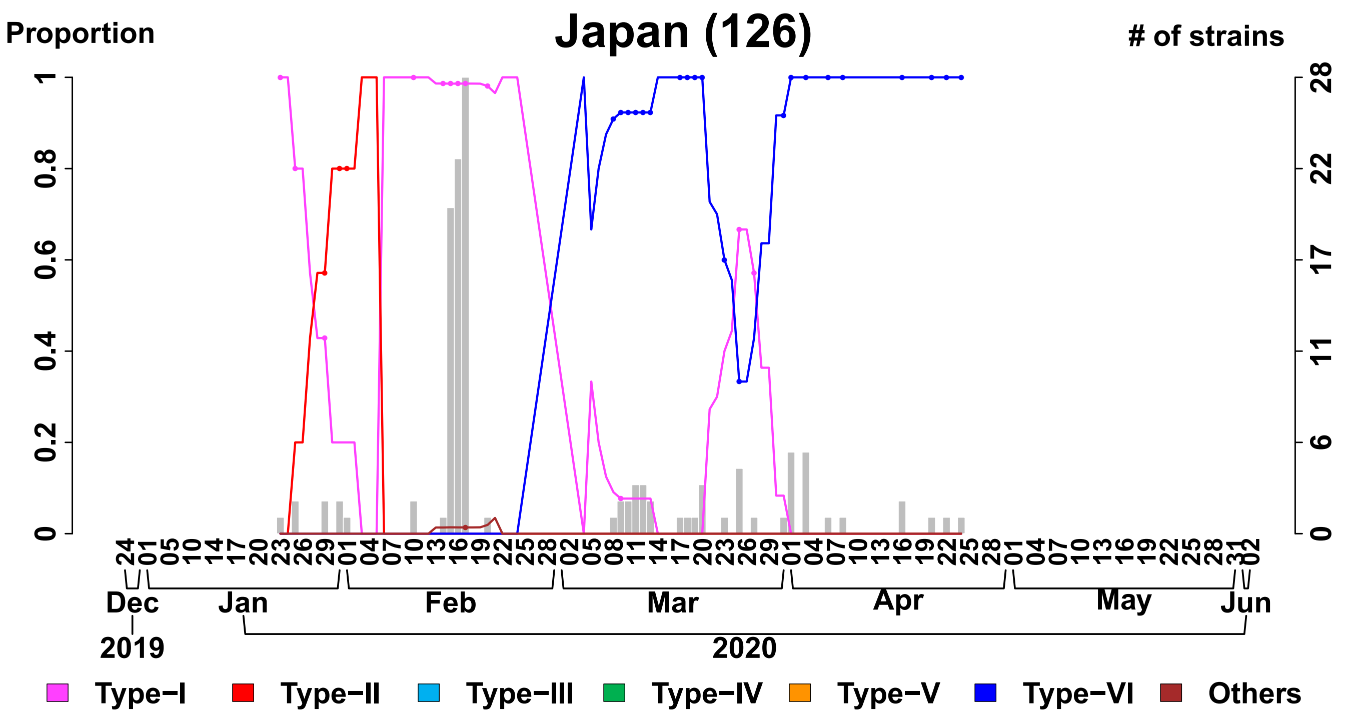

Fig. S4Y.

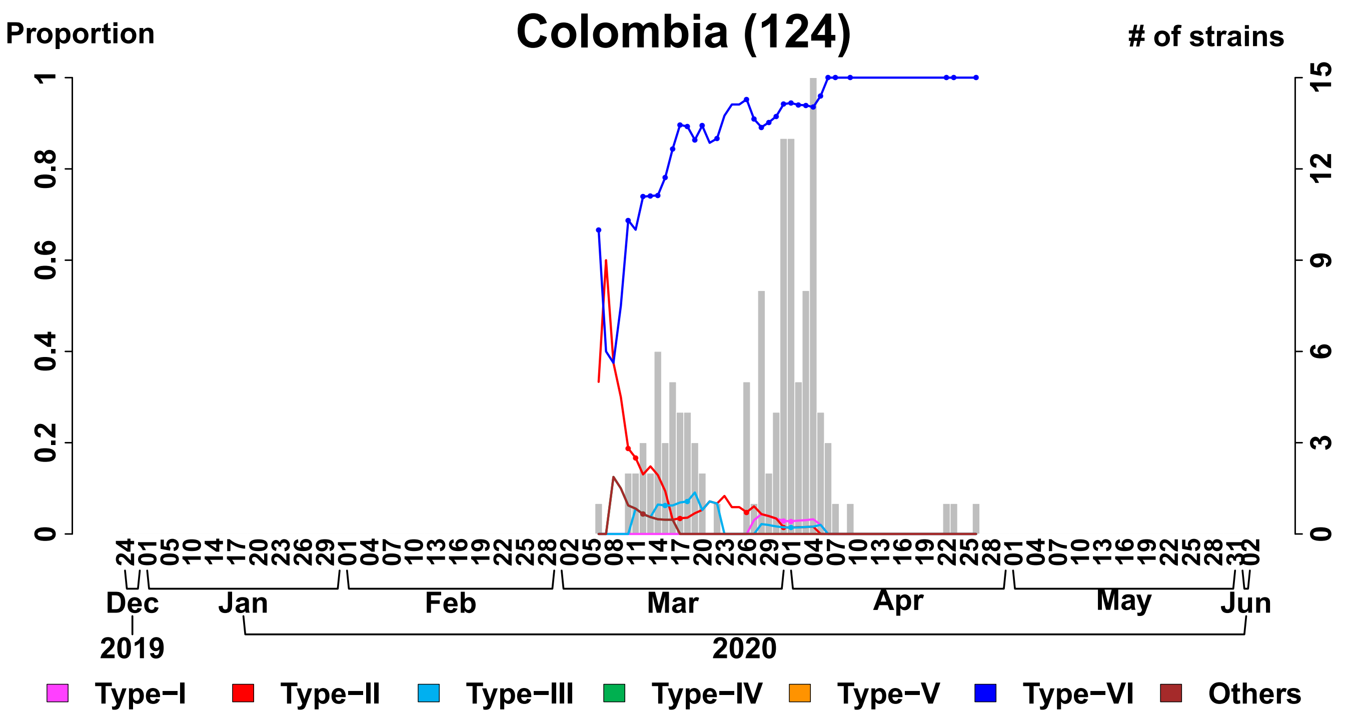

Fig. S4Z.

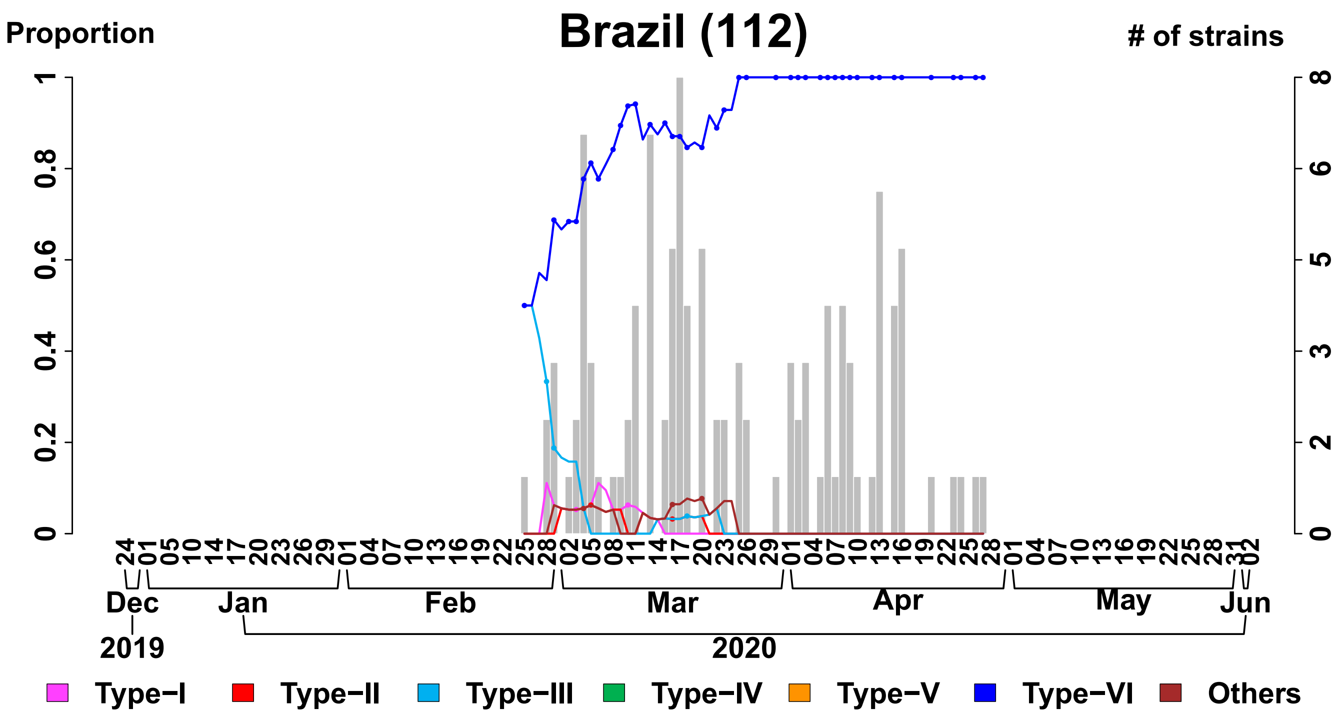

Fig. S4!.

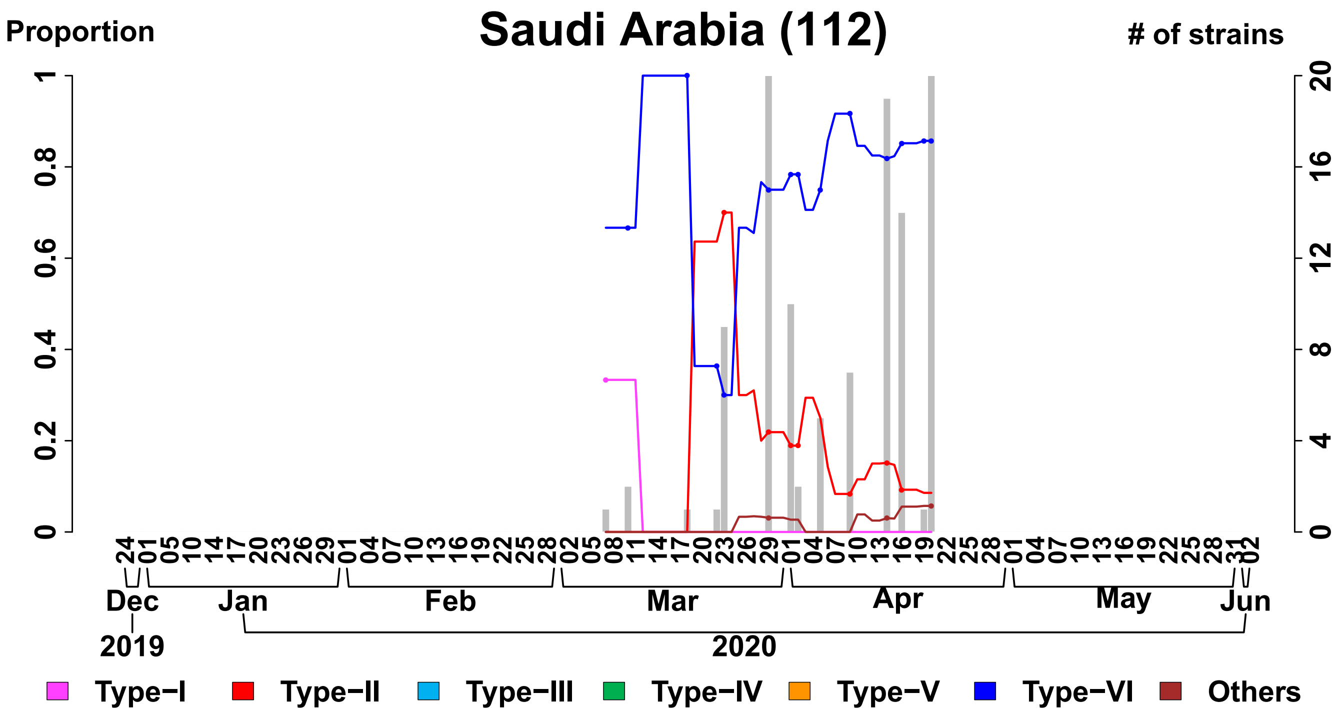

Fig. S4@.

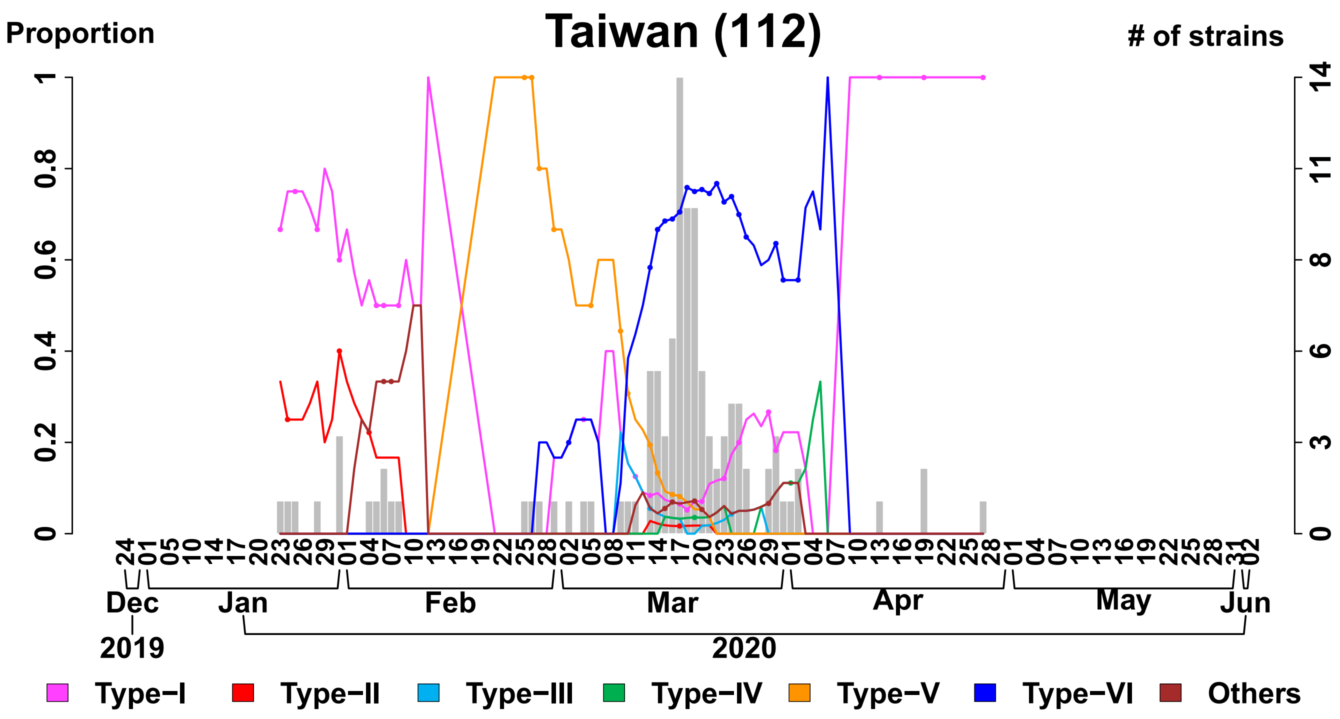

Taiwan (112)
Proportion
# of strains
0
0.2
0.4
0.6
0.8
1
0
3
6
8
11
14
Dec
Jan
Feb
Mar
Apr
May
Jun
2019
2020
Type−I
Type−II
Type−III
Type−IV
Type−V
Type−VI
Others

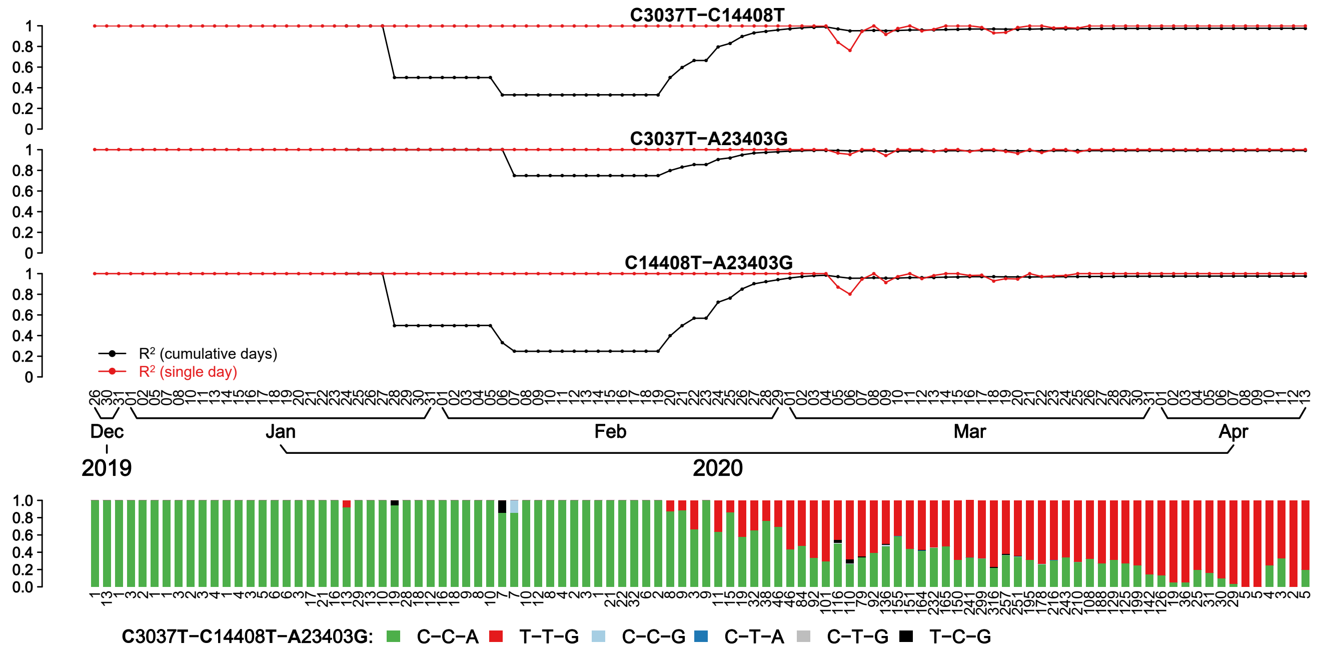

Fig. S5. Temporal pattern of pairwise allelic association for the Type VI signature SNVs C3037T, C14408T, A23403G and their haplotype frequencies. Pairwise allelic association (R^2) of the three Type VI signature SNVs was calculated based on the daily data (red dotted curve) or the cumulative data (black dotted curve). Haplotype frequencies were estimated based on the samples per date.

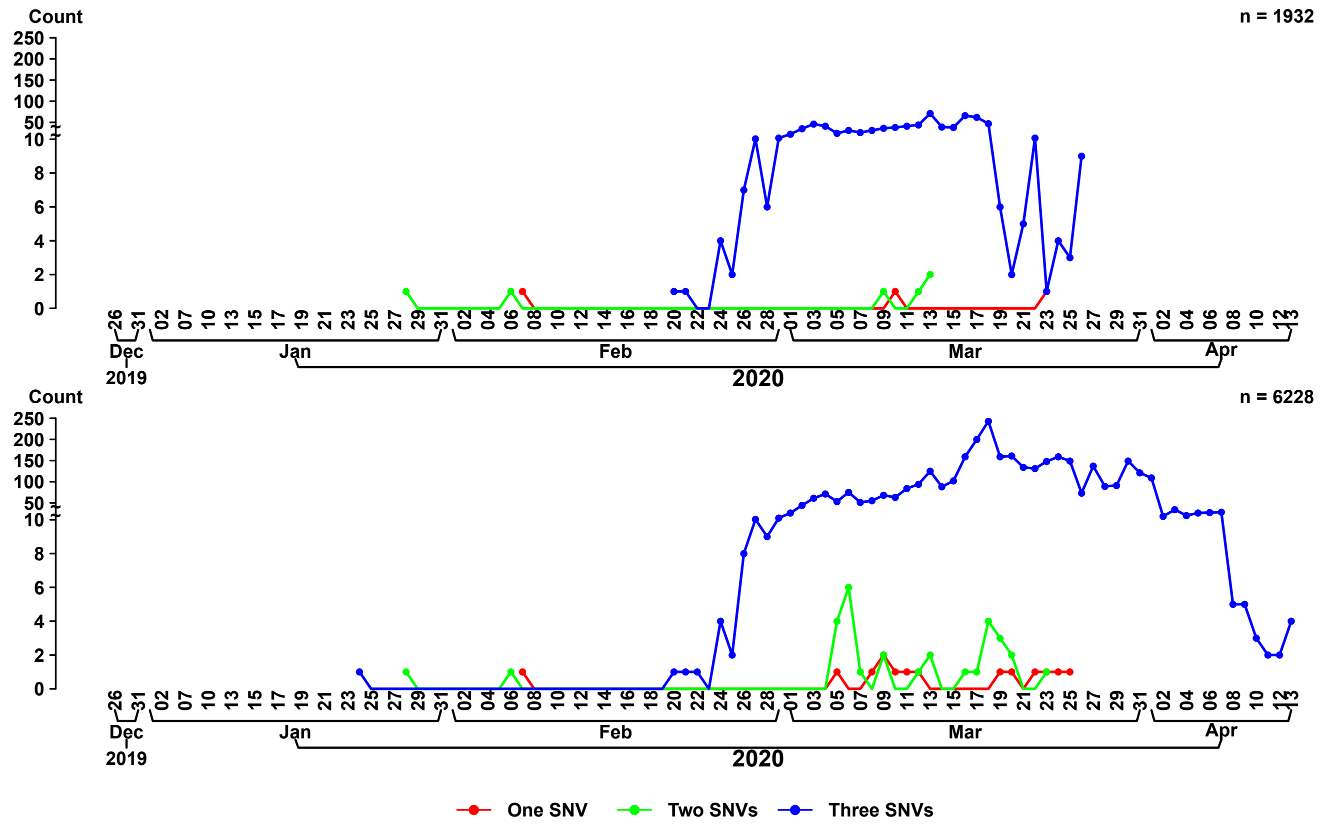

Fig. S6. Temporal trends of the occurrence counts for three, two, and one of the Type VI signature SNVs. The subfigures in the top and bottom show the trends of the occurrence counts in the data set of n = 1,932 and n = 6,228, respectively. In each subfigure, the trend of occurrence counts of three (blue line), two (green line), and one (red line) signature SNVs are displayed. The data set of 1,932 SARS-CoV-2 genomes showed that the TTG signature SNVs of Type VI appeared sequentially starting from Feb 20, 2020. The data set of 6,228 SARS-CoV-2 genomes showed that the TTG signature SNVs of Type VI first co-appeared on Jan 24, 2020.

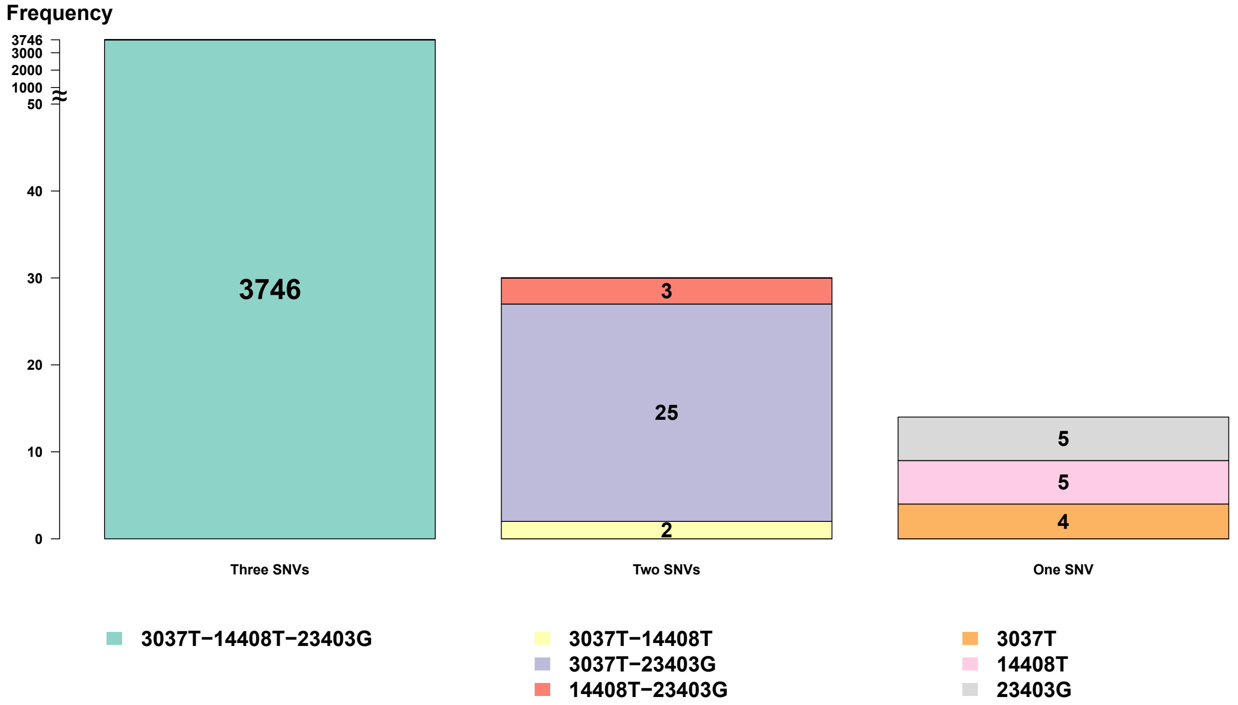

Fig. S7. Occurrence counts of three, two, and one of Type VI signature SNVs. The occurrence counts of three (left-hand-side bar), two (middle bar), and one (right-hand-side bar) of Type VI signature SNVs in the data set of n = 6,228.

Fig. S8A.

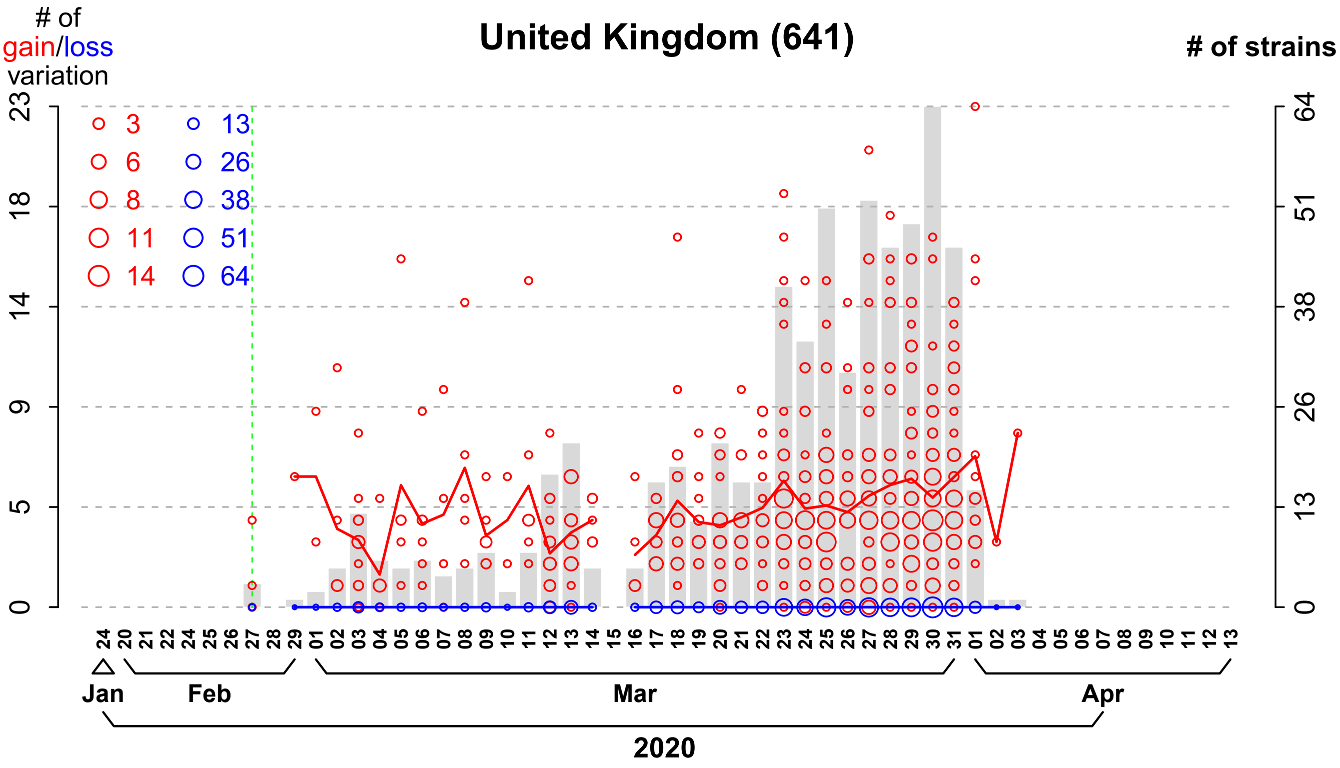

Fig. S8. Number of variation gain and loss in the Type VI strains in the nations that contributed a significant sample size. The left-hand-side vertical axis indicates the numbers of variation gain and loss. We picked one at random from the strains in the first date of sample collection in a country as a reference strain. Compared with the first strain in the country, in addition to the TTG signature SNVs, the Type VI strains in the country may have additional variations. Blue circles indicate the number of the strains that they lost the additional variations. Red circles indicate the number of the strains that they gained variations not in the reference strain. The larger circle represents the larger number of the strains that they lost/gained the additional variations. Sample size is displayed with a histogram in the background.

Fig. S8B.

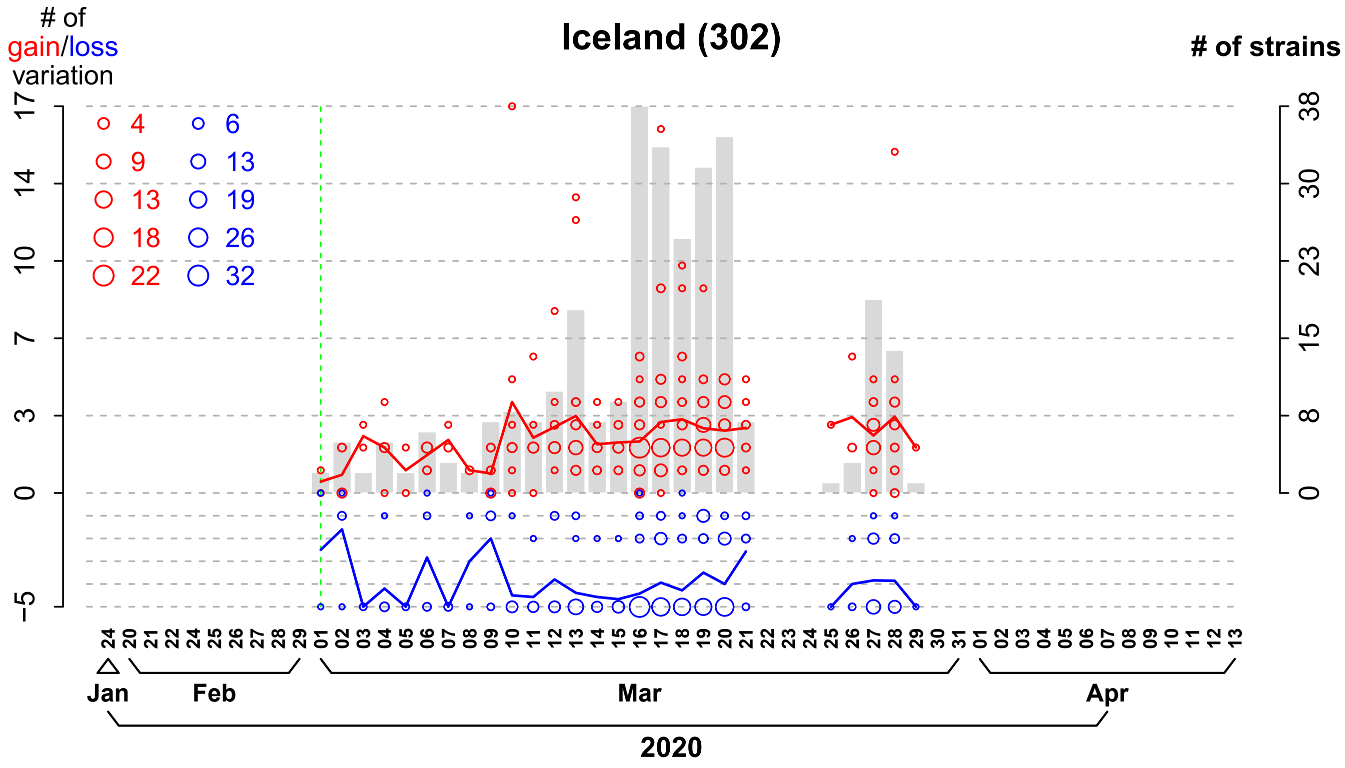

Fig. S8C.

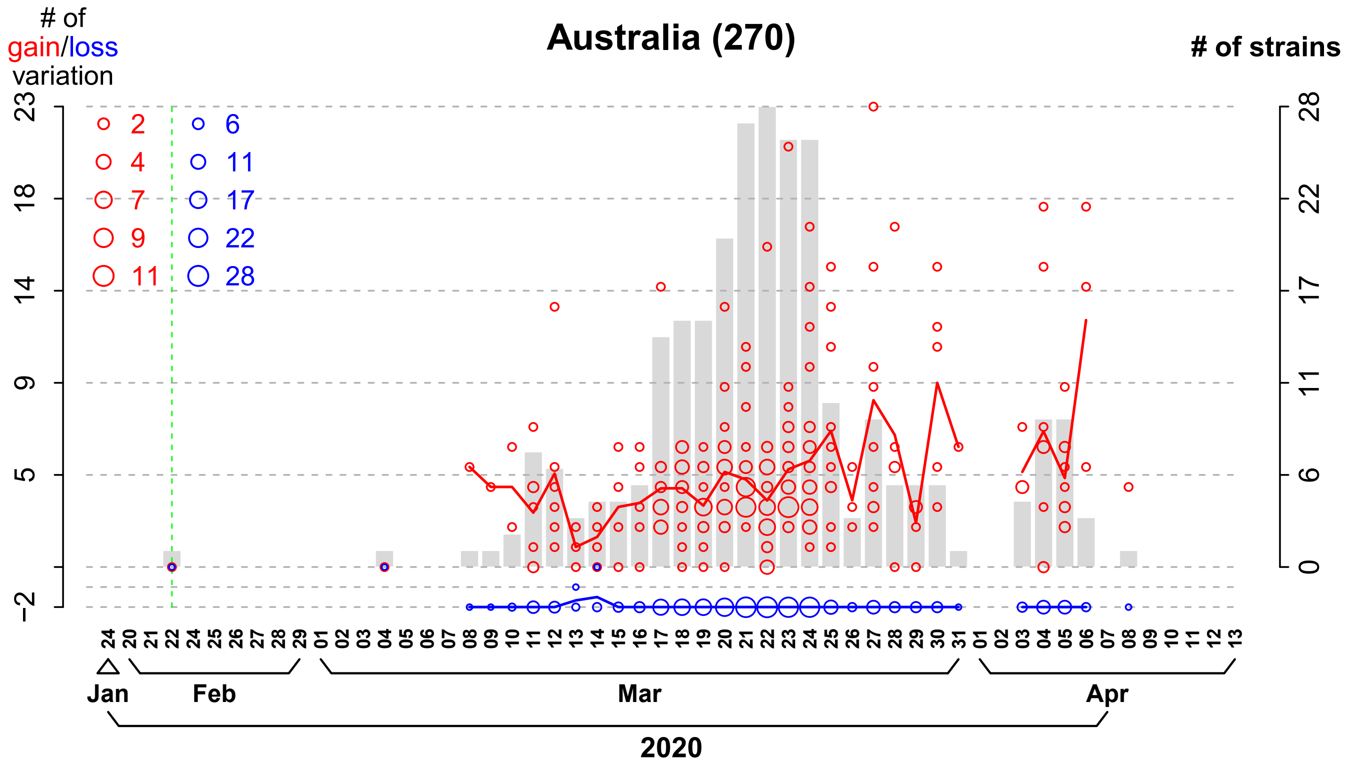

Fig. S8D.

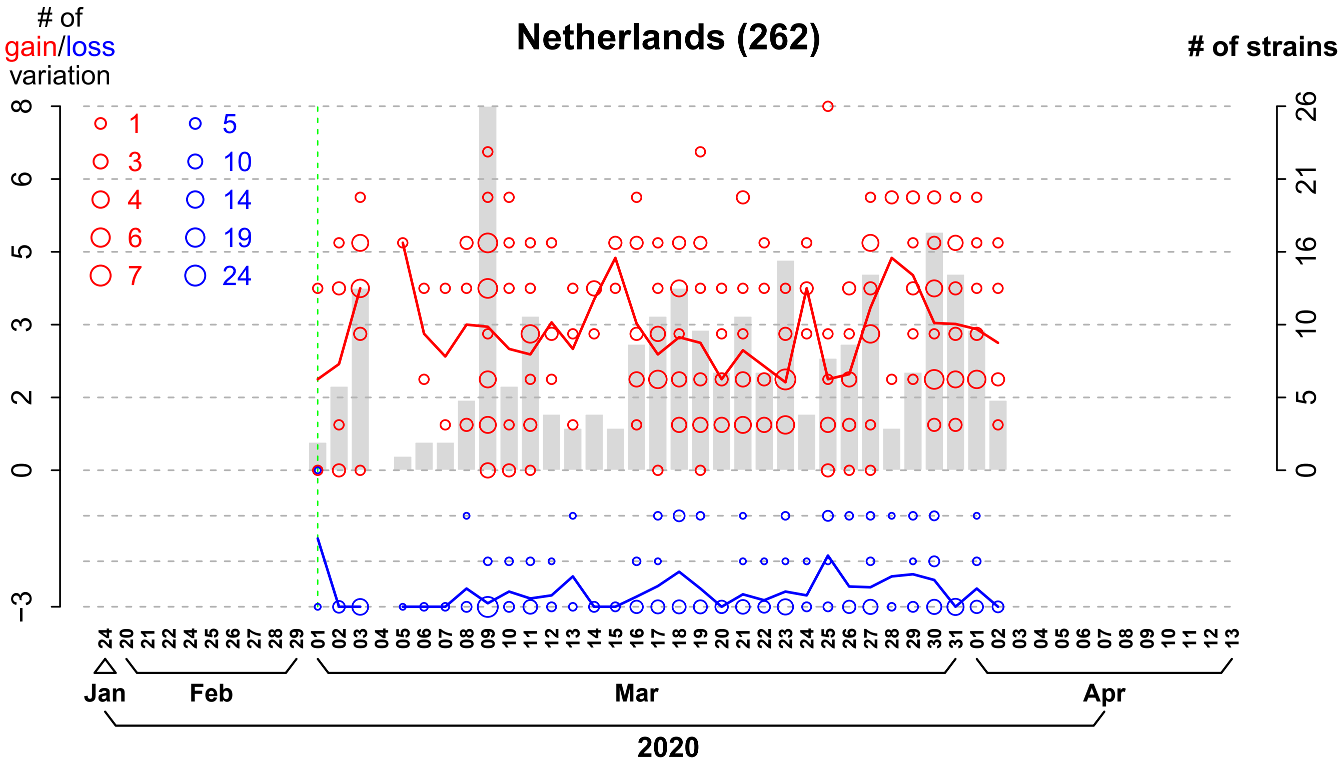

Fig. S8E.

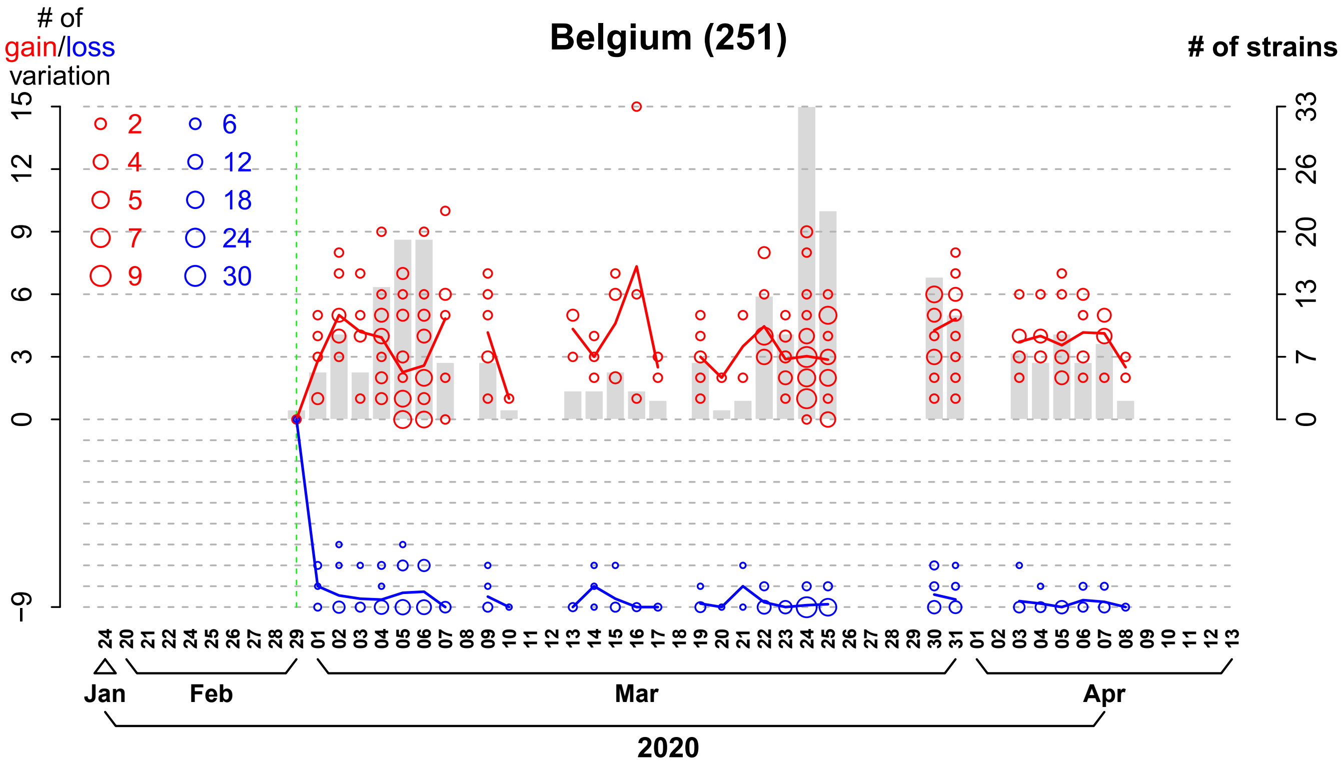

Fig. S8F.

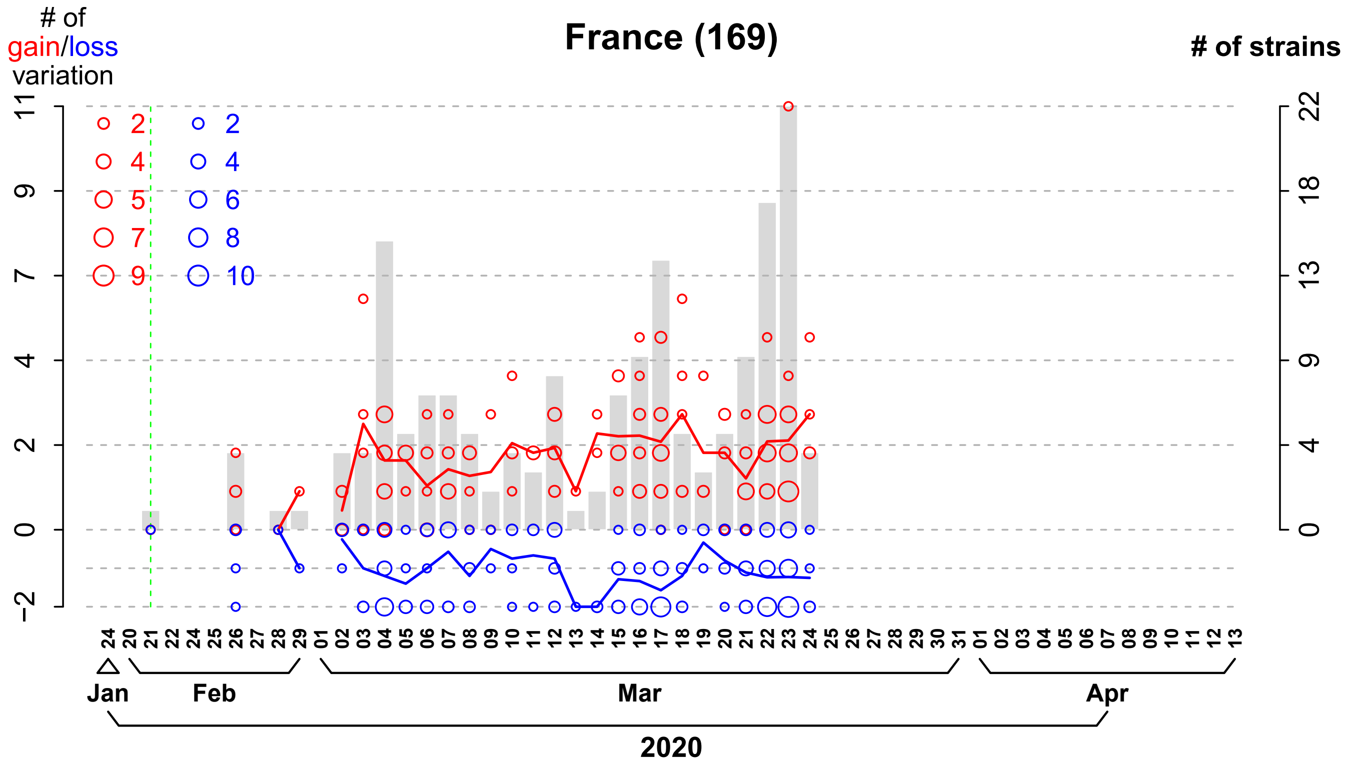

Fig. S8G.

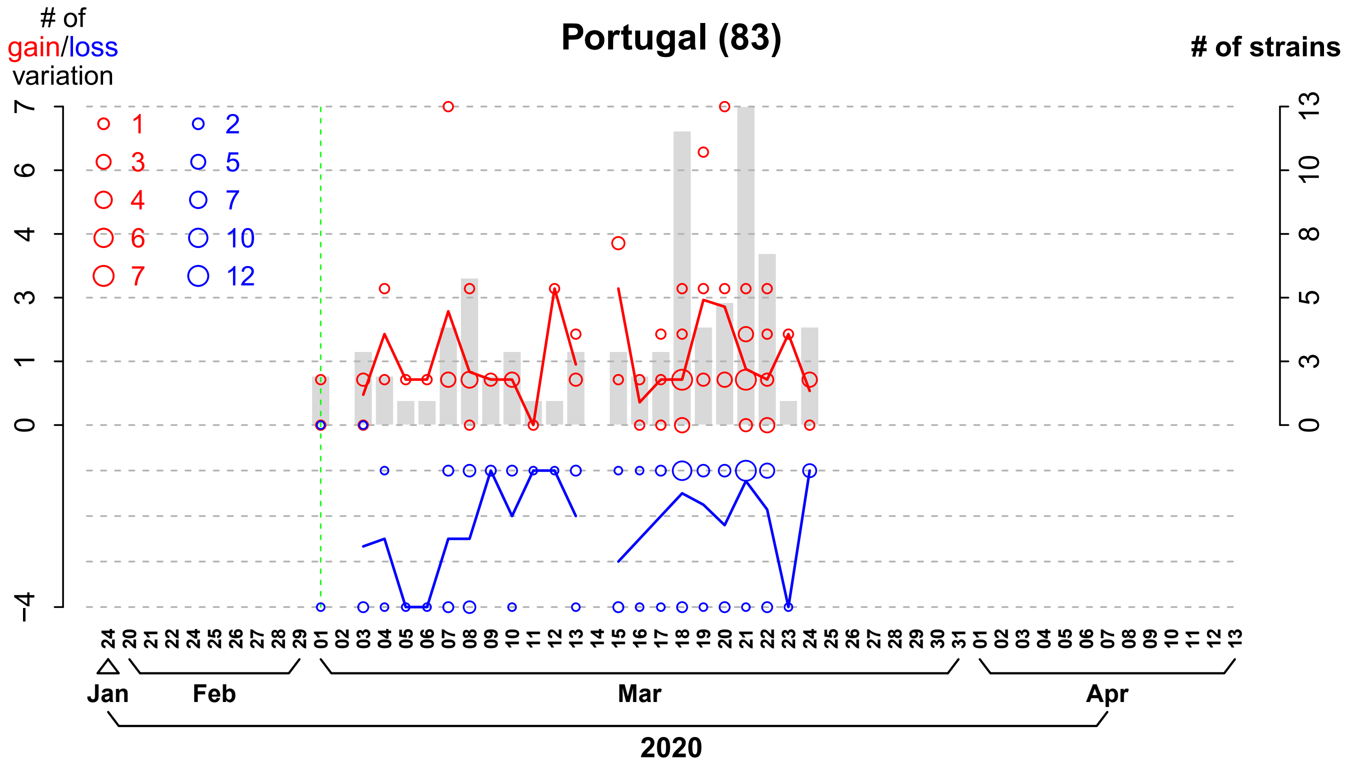

Fig. S8H.

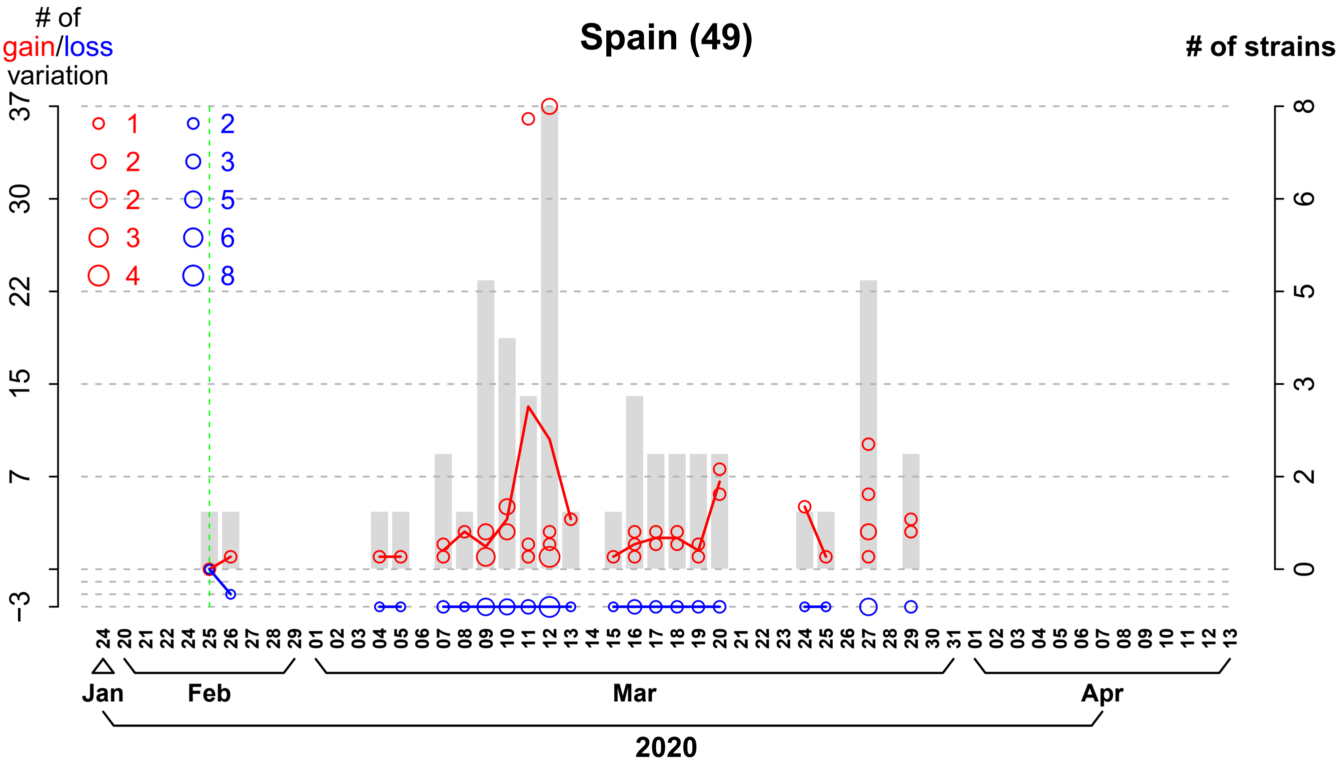

Fig. S8I.

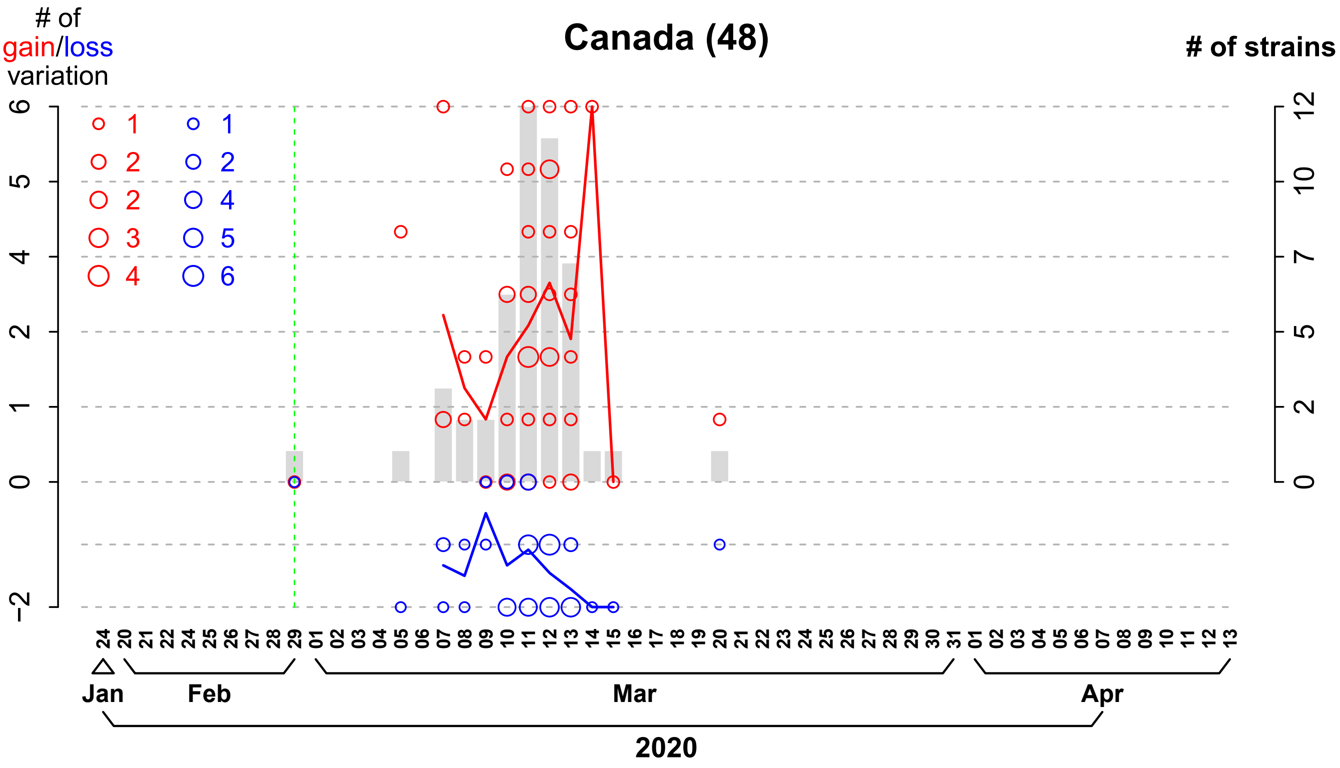

Fig. S8J.

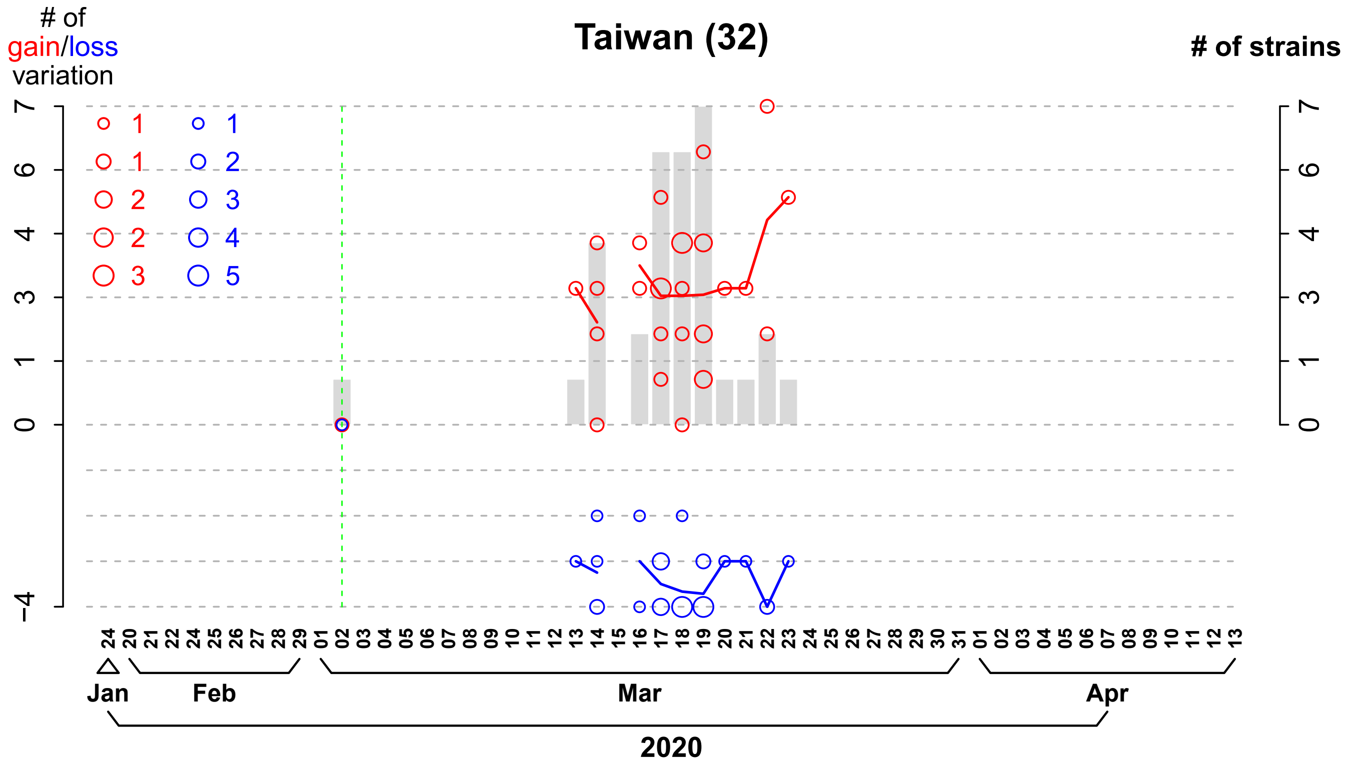
Taiwan (32)
of
gain/loss
variation
# of strains
Jan
Feb
Mar
Apr
2020

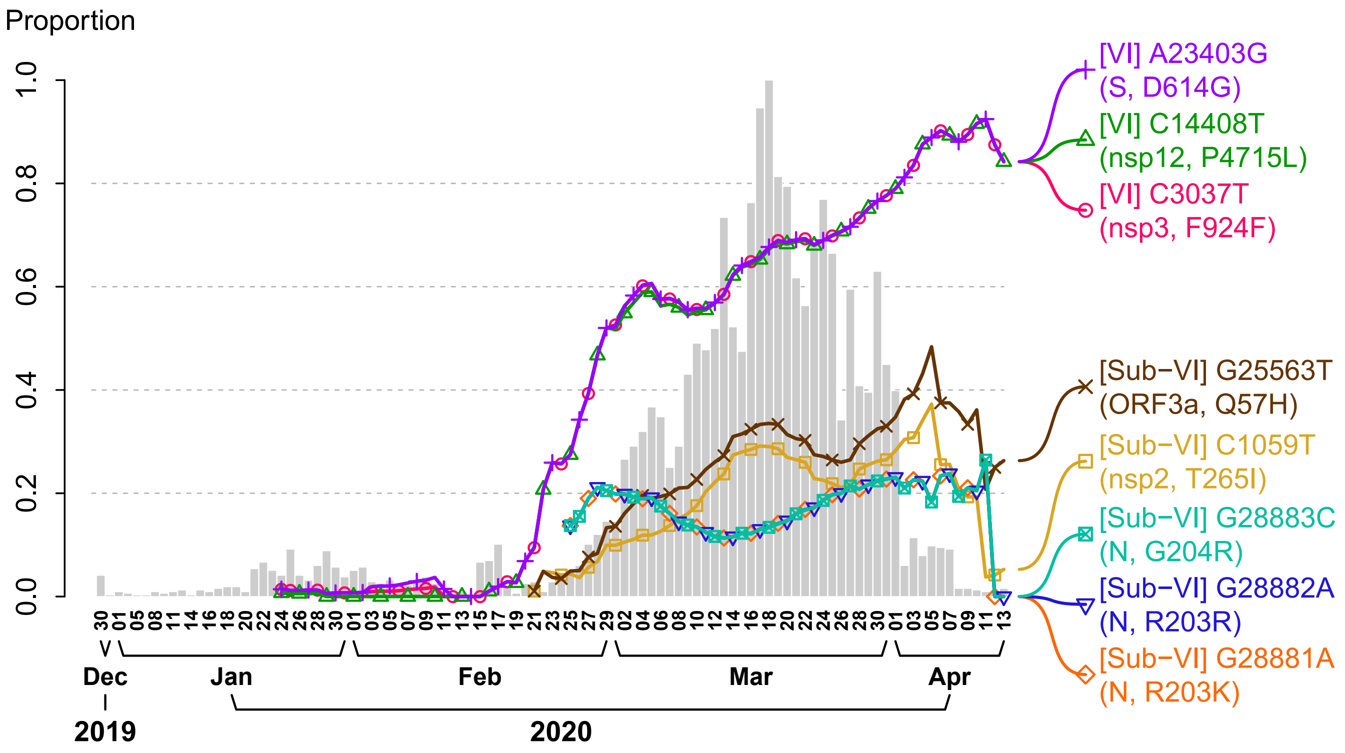

Fig. S9. Temporal variation frequency of the signature and sub-type SNVs. For a signature or sub-type SNV, the moving-window proportion on a sample collection date was calculated by dividing the number of signature SNVs (e.g., allele G for A23403G) by the number of the strains within four dates of the specific date on each side. Sample size is displayed with a histogram in the background.

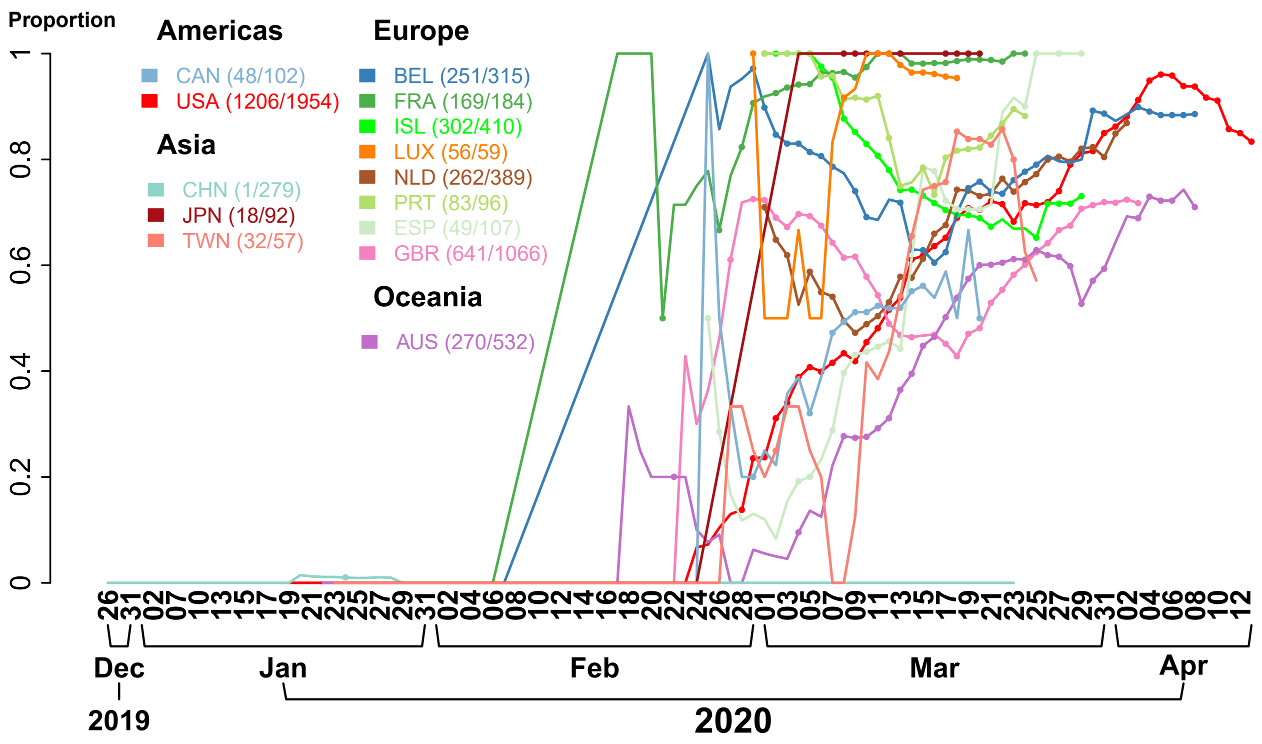

Fig. S10. Proportions of the Type VI strains in countries. The moving-window proportion was calculated by dividing the number of the Type VI strains by the total number of the strains within four dates of the specific date on each side in each country.
